# A dual-core NMR system for field-cycling singlet assisted diffusion NMR

**DOI:** 10.3389/fchem.2023.1229586

**Published:** 2023-07-05

**Authors:** Thomas B. R. Robertson, Rose C. Bannister, Topaz A. A. Cartlidge, Thimo Hugger, Sebastien Breham, Klaus Zick, Frank Engelke, Sam Thompson, Giuseppe Pileio

**Affiliations:** ^1^ School of Chemistry, University of Southampton, Southampton, United Kingdom; ^2^ Bruker Biospin GmbH, Silberstreifen, Rheinstetten, Germany

**Keywords:** singlet assisted diffusion NMR, long-lived spin order, diffusion NMR, NMR equipment, field cycling NMR

## Abstract

Long-lived singlet spin order offers the possibility to extend the spin memory by more than an order of magnitude. This enhancement can be used, among other applications, to *assist* NMR diffusion experiments in porous media where the extended lifetime of singlet spin order can be used to gain information about structural features of the medium as well as the dynamics of the imbibed phase. Other than offering the possibility to explore longer diffusion times of the order of many minutes that, for example, gives unprecedented access to tortuosity in structures with interconnected pores, singlet order has the important advantage to be immune to the internal field gradients generated by magnetic susceptibility inhomogeneities. These inhomogeneities, however, are responsible for very short T_2_ decay constants in high magnetic field and this precludes access to the singlet order in the first instance. To overcome this difficulty and take advantage of singlet order in diffusion experiments in porous media, we have here developed a dual-core system with radiofrequency and 3-axis pulsed field gradients facilities in low magnetic field, for preparation and manipulation of singlet order and a probe, in high magnetic field, for polarisation and detection. The system operates in field-cycling and can be used for a variety of NMR experiments including diffusion tensor imaging (both singlet assisted and not). In this paper we present and discuss the new hardware and its calibration, and demonstrate its capabilities through a variety of examples.

## Introduction

Molecular diffusion is encoded in a variety of magnetic resonance methods to extract structural and chemical-physical information on the diffusing molecule, the liquid it is dissolved in and the structure within which molecular diffusion occurs. Widely used examples of diffusion NMR experiments include: diffusion ordered spectroscopy (DOSY) where the signals belongings to the same molecular species in a mixture are separated and resolved according to their different diffusion coefficient (Morris, 2009); diffusion weighted MRI (DW-MRI) where contrast between tissues in the living matter is achieved through changes in the apparent diffusion coefficient of water confined within the different compartments characterising the micro-structure of specific tissues (Merboldt et al., 1985); diffusion tensor imaging (DTI) where the size, shape and orientation of compartments in a porous structure is mapped through the measurement of the whole diffusion tensor (Basser et al., 1994).

When molecules diffuse within a porous structure, measurements of the diffusion tensor provide structural information such as porosity, pore size distribution, tortuosity, etc., to characterise the porous medium itself. Such information is of fundamental importance to understand the property of the medium and/or guide the design of improved media (Callaghan, 2011). The anisotropic confinement of molecular diffusion, imposed by the structural characteristics of the medium, imparts a particular *shape* to the diffusion tensor and this can be measured through the DTI technique. DTI provides a form of indirect imaging of structures with pores that are too small for real-space magnetic resonance imaging (this latter has a typical spatial resolution of ∼0.1–1 mm^3^). In structures with relatively big pores (say above 100 *μ*m^3^), for which structural imaging via MRI can already provide good quality 2D and 3D images, diffusion-NMR techniques would still play an important role in its ability to catch the dynamics inside the medium, an important information that is fundamental to many applications. For example, the connectivity between distant pores, as rendered by the tortuosity parameter, is of crucial importance in battery electrodes and fuel-cells gas diffusion layers. Similarly, tortuosity is relatable to the availability of nutrients and the removal of waste in the various parts of scaffoldings used in tissue engineering. Generally, if the pores of a medium are too big with respect to the maximum distance traveled by molecules during the experiment, then the anisotropic confinement is not correctly captured and the structure within which molecules diffuse appears to be (incorrectly) isotropic, making DTI-derived information unreliable. This becomes relevant considering that, in conventional diffusion NMR experiments, the molecular diffusion time is limited by the lifetime of either transverse or longitudinal nuclear spin order. Most typically, the lifetime of longitudinal order is bigger or equal than the lifetime of transverse spin order, ranging between a few milliseconds and a few seconds. The relatively short persistence of diffusion-encoded NMR signals translates in a limitation to the size of pores and pore-pore distances that can be reliably probed via conventional diffusion-NMR techniques. However, in some circumstances, like, for example, when dealing with low-gamma nuclei such as ^13^C or ^15^N to cite a few commonly-encountered species, the lifetime of longitudinal and transverse spin order can be of the order of many tens of seconds or even a few minutes. Systems possessing such long spin order lifetimes can be therefore used as spies to probe anysotropic confinement, pore interconnectivity and so on.

Working on the same logic but using a different approach, our group has recently proposed to extended the scope of diffusion-NMR through the use of long-lived singlet spin order (Dumez et al., 2014; Pileio et al., 2015; Pileio and Ostrowska, 2017; Tourell et al., 2018). In two-spin-1/2 systems and under well-understood circumstances, singlet order persists for many minutes (sometimes many tens of) against the few-seconds-long persistence of longitudinal or transverse order prepared in the same systems (Pileio, 2020). Long-lived singlet order can be generated through a variety of pulse sequence schemes (Pileio, 2017) and, by combining singlet order preparation/readout schemes with diffusion encoding pulsed field gradient techniques it was possible to measure small diffusion coefficients (Cavadini et al., 2005; Cavadini and Vasos, 2008), slow dynamic processes, (Sarkar et al., 2007a; Sarkar et al., 2007b), slow flows, (Pileio et al., 2015), cavity sizes of the order of millimetres through singlet enhanced q-space imaging (Yadav et al., 2010; Torres et al., 2012; Pileio and Ostrowska, 2017), track molecules over minute-long time intervals (Dumez et al., 2014), and measure shape and orientation of millimetre-sized channels in porous media via singlet assisted DTI (paper in preparation). We have dubbed the generic class encompassing all these techniques as singlet-assisted diffusion NMR (SAD-NMR).

However, the analysis of diffusion NMR data in porous structures is often complicated by phenomena related to the magnetic susceptibility mismatch between the porous matrix and the imbibing liquid (or gas). These susceptibility differences, despite often just of the order of a few ppm’s, create two sorts of deleterious problems, they: (i) generate internal field gradients whose intensity is often larger than the field gradient pulses used to encode molecular diffusion in NMR; (ii) produce a strong relaxation mechanism for transverse magnetisation (Callaghan, 1991; Callaghan, 2011). With regard to the first problem, SAD-NMR is very advantageous since singlet order is immune to magnetic field gradients. However, the strong transverse magnetization decay in porous media impedes the preparation of singlet order because this typically requires transverse magnetisation to survive for tens (sometimes hundreds) of milliseconds. In order to understand this phenomenon, some of us have recently developed analytical equations and a simulation code to predict the relaxation of transverse order due to susceptibility inhomogeneities in a porous structure of arbitrary complexity (Cartlidge et al., 2022). The severity of these effects depends upon the value of the static magnetic field the sample is immersed in, and become negligible at magnetic field strengths of the order of 100 mT or below, depending on the actual size of the inhomogeneities. Unfortunately, magnetic resonance detection at such low field is very poor and only time-domain NMR experiments are then feasible. In time-domain however, chemical shift resolution is lost, with all related consequences.

The use of low magnetic field, required to reduce susceptibility-related issues, is here combined with the high-resolution and high-sensitivity features of high-field NMR by working in a field-cycling fashion. Field-cycling NMR has become an active area of magnetic resonance with application that spans from gathering dispersion curves (measurement of relaxation at different magnetic fields) for studying food, proteins and MRI contrast agents, to gaining contrast in low-field MRI experiments (Anoardo et al., 2001; Kimmich and Anoardo, 2004; Broche et al., 2019). Field-cycling can be implemented in two complementary ways: (i) by ramping the magnetic field with the use of an electromagnet or, (ii) by shuttling the sample between two (or more) regions of space with different magnetic field values. The first method provides a very rapid field-switching time (a Tesla in a few milliseconds), but the maximum field achievable is limited to a relatively low value of around 2 Tesla. The sample shuttling method is somewhat slower (although some shuttle systems can cover many Tesla within tens of milliseconds) but can be implemented around virtually any available static magnetic field value. Several groups have built sample shuttles to run magnetic resonance experiments at two or more fields (Swanson and Kennedy, 1993; Redfield, 2003; Chou et al., 2012; Charlier et al., 2013; Cousin et al., 2016; Zhukov et al., 2018; TomHon et al., 2020). Our laboratory has a custom-built sample shuttle to measure relaxation of longitudinal (T_1_), transverse (T_2_) and singlet order (T_S_) and a temperature-controlled sample shuttle where the sample temperature is maintained constant through the sample within 0.05°C as the sample travels across magnetic field spanning from 7 T to 50 mT (Hall et al., 2020).

In this paper, we report about the construction of a dual-core NMR system with radiofrequency facilities at both 7 T and 46.4 mT (500 kHz ^13^C Larmor frequency) plus 3-axis gradient facilities at 46.4 mT to allow singlet-assisted diffusion tensor imaging in porous media (and several other experiments, including SAD-NMR schemes) at a field where the negative effects of magnetic susceptibility inhomogeneities become negligible. The system is complemented by a shuttle with 3-axis accurate sample positioning that moves the sample from the high-field probe, where the sample is firstly polarised and later detected, to the low field probe where diffusion is encoded into singlet order via a combination of pulsed field gradients and radiofrequency pulses. The paper aims to discuss the details of the equipment and demonstrates its uses by measuring T_1_, T_2_, T_S_, diffusion coefficients, tortuosity and diffusion tensors in isotropic liquid samples as well as in porous media with large magnetic susceptibility inhomogeneities.

## Hardware development

### Hardware design

**FIGURE 1:**
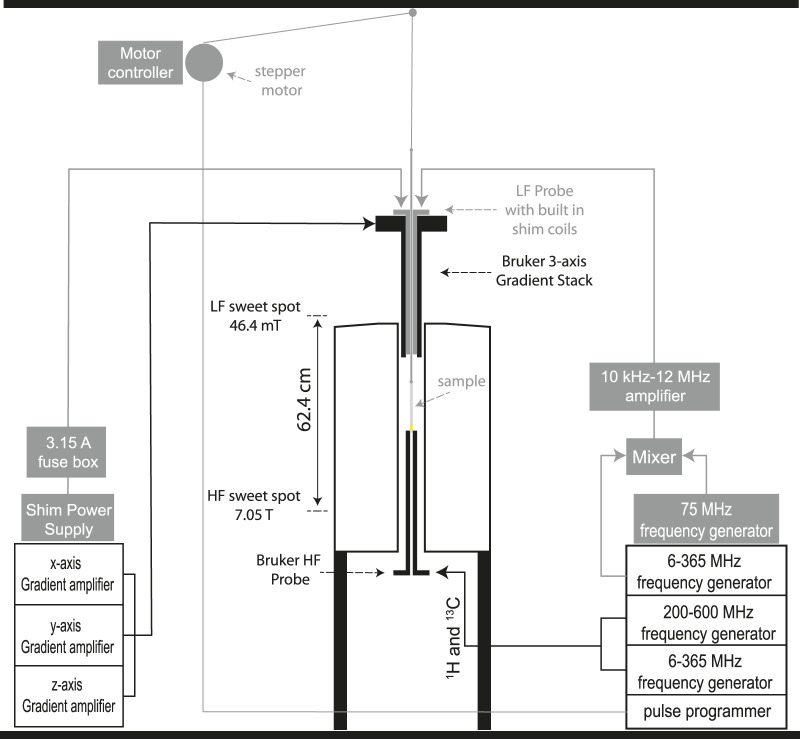
A schematic view of the dual-core magnetic resonance setup developed in this work. All parts in grey are customised add-ons to existing hardware.

**FIGURE 2:**
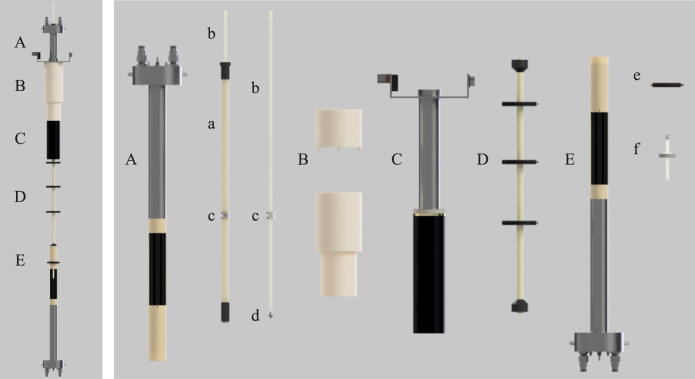
A rendered view of the setup developed in this work. **(A)** custom-made 500 kHz probe, **(B)** POM mounting for probe/gradients onto top of magnet bore, **(C)** Bruker Micro 2.5 WB 3-axis gradients, **(D)** Sample guide tube connecting low and high field probes, **(E)** Bruker MICWB40 probe with custom centering guide and sample depth gauge (e, f). Internal components are: (a) GRP sample rod guide tube with xy rod guide (labelled as c); (b) ASA 7 mm square sample guide rod; (c) Perspex rod guide cylinder with 7.1 mm square hole to ensure sample remains coaxial with both probes and is consistently positioned within the xy plane; (d) Acrylic attachment with external M5 screw thread for NMR tube mounting; (e) Centering guide for high field probe mounting; (f) PTFE sample positioning stopper for shuttle.

The customised equipment here presented is built and assembled around a 7.05 T Oxford Instruments unshielded magnet coupled to a Bruker Avance III 300 MHz console and equipped with a 10 mm MICWB40 Bruker probe with a ^1^H/^13^C resonator that sits in the 7.05 T sweet spot (Figure 2E). A schematic view of the setup is reported in Figure 1 with the actual parts rendered in 3D in Figure 2. More details about the construction are reported in the Supplementary Material to this paper. The regions labelled as high (HF) and low field (LF) correspond to 7.05 T and 46.4 mT, respectively. These are located in the magnet sweet spot (HF) and 62.4 cm above the sweet spot along the magnet stray field (LF). At 46.4 mT the Larmor frequency of ^13^C is 500 kHz which is the frequency at which the LF probe is tuned. The next few sections contain details of the hardware setup.

### Low field probe

The LF probe (Figure 2A) was designed and manufactured in collaboration with Bruker BioSpin GmbH. The circuitry is built inside a modified MICWB40 Bruker probe case which is bore-through to facilitate the sample shuttling stage. The probe radiofrequency coil is tuned to 500 kHz and accommodates a 10 mm NMR tube. It is a saddle coil with 10 mm inner diameter and 30 mm length. The B_1_ field has been calculated to be 0.12 mT A^−1^ with a quite flat profile over 20 mm. The coil center is placed in a region of the stray field where the maximum field spread over 20 mm has been measured to be 4.7 mT, corresponding to about 50 kHz for ^13^C. Therefore, the probe is also equipped with a Z-shim coil to correct for these inhomogeneities. The shim coil geometry is optimized for linearity in a cylindrical volume with 10 mm diameter and 25 mm length and was wound with copper wire with a diameter of 1 mm.

Special care had to be taken regarding the RF coil performance. A design goal of a conventional NMR coils is usually to maximize the Q-factor for maximum transmit and receive efficiency, but due to the very low working frequency of 500 kHz, a high Q-factor would result in a very long rise time of the current in the resonant circuit, which would limit the achievable width of the excitation profile, due to the required short pulse lengths of the experiments. On the other hand, reducing the Q-factor too much would result in mean and peak power values that are just not feasible due to the non-availability of amplifiers and of course electronic failure of the probe due to arcing and heating. Therefore, a compromise between a fast rise time and low reference power had to be found. We therefore used both Electromagnetic and spin dynamics simulations to found out that a saddle coil with 4 windings and an added resistance would constitute a good compromise with a calculated Q-factor value of about 5 (see Supplementary Material).

### 3-axis field gradients

The low field probe fits within the 40 mm internal diameter of a Bruker Micro2.5 WB 3-axis gradient system (Figure 2C). Gradients are driven by a Bruker GREAT 60 A amplifier rack generating a maximum gradient strength of 1.5 T m^−1^. The gradient system is held inverted and centred in the LF spot by a custom built plastic support detailed in Supplementary Figure S5.

### Sample shuttle

Sample transport between the HF and LF sweet spots is achieved using a Trinamic TMCL-1160 stepper motor operated by the customised software as previously reported by our group (Hall et al., 2020). The motor is positioned outside the magnet stray field at ceiling height. Unfortunately, due to limited ceiling clearance and the need for the sample guide rod described below, it was not feasible to mount the stepper motor directly above the magnet as would be ideal. A 25 cm circumference spindle wheel 3D-printed from ABS plastic winds a high tensile strength Dyneema cord with a low stretch ratio. The cord pulls up and down an acrylonitrile styrene acrylate (ASA) 70 cm long sample guide rod with a 7 mm × 7 mm square section profile and 1 mm wall thickness (part b in Figure 2). The rod slides through a square hole of 7.1 mm size (Figure 2C), placed within the LF probe body in order to maintain micrometric sample alignment at all time as necessary for DTI structural studies. The top of the square rod engages via a brass hook to the pulling cord. The bottom of the square rod has an acrylic attachment (part d in Figure 2) with an external (M5) screw thread to allow attachment to a standard 5 or 10 mm Wilmad-Labglass Pressure Vacuum Valve NMR Tubes for rapid sample exchange between experiments. The shuttle’s speed and its acceleration profile can be adjusted and specified in the spectrometer’s user interface (Bruker Topspin’s interface in our case). We have also implemented the modality in which the user specifies the shuttling time and the velocity is adjusted accordingly. Our intended use of this shuttle gravitates around long-lived spin order in doubly-labelled ^13^C molecules. Typically, these molecules have many-second long T_1_ and thus fast shuttling times are not needed. In a typical experiment we use a shuttling time of 3 s to cover the HF-LF distance of 62.4 cm, corresponding to about 0.2 m s^−1^.

### Electronics

The 500 kHz radiofrequency is generated by mixing the signal generated by one channel of the Bruker console, set at 75.5 MHz, with the one generated by an external frequency synthesizer (PTS 250SHO2EYX-8/X-26), set at a fixed frequency of 75.0 MHz and synchronised on the same clock of the Bruker console. Frequency mixing is done using a Mini-circuits ZP-3-S+ frequency mixer. The output signal is filtered via a Mini-circuits BLP-70 low pass filter to eliminate the higher frequency. The so-generated signal is amplified by a 40 W RF Amplifier working in the range 10kHz-12 MHz at 50 dB (purchased from Electronics and Innovation, model 2100L).

The Z-shim coil within the LF probe is powered by a Rohde and Schwarz NGA101 power supply. The current reaching the Z-shim coil is limited to 5 A with the use of a fuse box placed along the transmission line.

A custom-printed circuit board is used to supply power to the motor driver and to amplify the trigger outputs from the spectrometer console (working at 5 V) to the motor controller (working at 10 V). Position and timing of the shuttle is controlled by the spectrometer computer using custom-made Python scripts integrated within the acquisition software. Prior to acquisition, the values for the speed, acceleration and target field strength are set by the user within the acquisition tab, read by the Python scripts and stored in the motor memory. During acquisition, TTL signals from the spectrometer console triggers the motor to move up or down at times specified in the pulse program.

## Hardware calibration

This section describes the experiments and procedures done to optimise the magnetic field homogeneity in the LF region and to calibrate the pulse length for ^13^C in the LF probe.

### Field shimming

To find the sweet spot for the LF probe, we measured the stray field above the sweet spot of the 7.05 T magnet and along the *z*-axis for over 1 m, and with a 1 cm spatial resolution, using a Hall device (Lakeshore 460 3 channel gaussmeter with MMZ-2518-UH probe). A 500 kHz Larmor frequency for the ^13^C would correspond to a field of 46.4 mT and this was found to be 62.4 cm above the HF sweet spot. The magnetic field within 10 mm above and below this point (the LF sweet spot) has been sampled with a 5 ± 1 mm spatial resolution (see column 2, Supplementary Table S1 in Supplementary Material) and was found to vary, almost linearly, by about 4.8 mT across the 20 mm region.

To obtain a ballpark value for the current to be supplied to the Z-shim coil placed within the LF probe to correct for the B_0_ inhomogeneity, we have measured the magnetic field around the LF sweet spot as a function of the current supplied. The results of these measurements are reported in columns 3-14 of Supplementary Table S1 in Supplementary Material. From these data, which suffer from imperfection in the manual positioning of the field probe (estimated to be of the order of 1 mm), the field results almost flat within 0.1 mT and over 20 mm when the Z-shim is driven by a 3.2 A current.

Ahead of these field measurements we have checked that the inner surface of the LF probe does not heat too much when the Z-shim coil is turned on. This has been done with the use of a PT100 temperature probe placed in the centre of the coil region while airflow through the coil was restricted. These measurements (Supplementary Figures S17, 18 in Supplementary Material) show that a 3.5 A current leads to an increase in temperature from 20.8 to a maximum of 39.5°C in 80 min.

**FIGURE 3:**
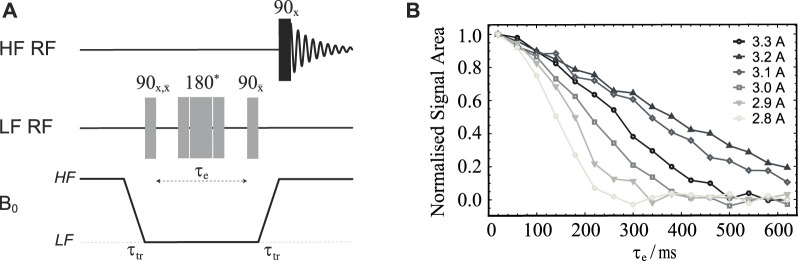
**(A)** Pulse sequence used to optimise the magnetic field homogeneity around the LF sweet spot. The central 180° pulse marked with an asterisk is a composite pulse implemented as 90_*x*_180_*y*_90_*x*_. The duration of the 90° pulse length was 21.5 *μ*s (obtained as explained below). **(B)** Normalised signal area plotted against the echo time *τ*_*e*_ obtained using the pulse sequence in **(A)** and sample **S1** (see Table 1). The different curves refer to experiments run with different values of electric current in the Z-shim coil (as shown).

Clearly, these shim adjustments ignore the sample and all the shuttle mobile parts. To obtain an actual value for the field homogeneity correction required for the complete system, we have measured the decay of transverse magnetization for sample **S1** as a function of the variable echo time using the field-cycling version of a spin-echo pulse sequence shown in Figure 3A applied for different values of the current supplied to the Z-shim coil.

Diffusion in a field gradient is, in fact, a well studied phenomenon and analytical equations are readily available (Callaghan, 2011; Cartlidge et al., 2022). These equations basically say that the better the field homogeneity the slower NMR signals will decay. This is shown in Supplementary Figure S29 of Supplementary Material, which reports about a simulation of the phenomenon using recently published methods (Cartlidge et al., 2022). Hence, for the sake of optimising the shim current, one can qualitatively observe which current produces the slower signal decay curve in a single echo experiment with variable echo times. The results of these calibration experiments, run on sample **S1** (see **Materials and Methods** section), for a set of Z-shim coil currents around 3 A, are reported in Figure 3B and show that the best shimming is achieved by supplying 3.2 A, as previously measured without the presence of the sample.

Finally, we have checked the sample internal temperature using a sample of ethylene glycol to find that the internal temperature of the sample while in LF is (30.5 ± 0.5) °C, in an experiment involving multiple scans with the longest time spent in low field (2 min, see Figure 11) and a 3 min wait in HF (see Supplementary Material). Note that the sample sits at 25°C while in the HF probe. The heating in LF is due to the heat produced by the shim coil. This can be drastically minimized if the shim corrections are turned *on* during the pulse sequence only and turned *off* while the sample is in HF or during the diffusion time in diffusion experiments.

### Pulse calibration

**FIGURE 4:**
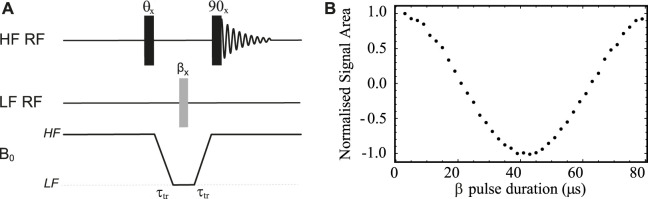
**(A)** Pulse sequence used to calibrate ^13^C pulse length in the LF probe. **(B)** Low field pulse calibration curves obtained using the pulse sequence in **(A)** and sample **S1** (see Table 1). The optimisation was obtained with a 3.2 A electric current supplied to the Z-shim coil.

To optimise the pulse length for ^13^C at 500 kHz Larmor frequency, we used a sample of ^13^C_1_ sodium pyruvate in D_2_O (sample **S1** in Table 1) and the pulse sequence shown in Figure 4A. In these experiments, two transients where collected at each value of the low field pulse duration (*β*) in order to compensate for magnetization build-up during transport between the low and the high fields. The first HF pulse is absent (*θ* = 0°) during the first transient whereas it becomes a 180° pulse for the second transient. Concomitantly, the receiver’s phase is cycled between 0° and 180° between the two transients, effectively subtracting the signal acquired in the two transients. Prior to pulse optimisation, and in order to find the correlation between the nominal and the effective power output of the amplifier, the peak-to-peak voltage produced by the low field amplifier was measured as a function of the spectrometer’s power level settings for the channel. Knowing the coil characteristics, we have decided to supply an effective power of 61.6 W, corresponding to a voltage of 55.5 V. According to simulation of the actual coil, this should provide a pulse length of around 20 *μ*s for a 90° pulse.

**TABLE 1:** Labeling and composition of samples used in this work.

name	Molecular spy	Solvent	Beads
**S1**	^13^C_1_ sodium pyruvate	D_2_O	-
**S2**	1,2-diphenyl-^13^C_2_-acetylene	CD_3_CN	-
**S3**	1,2-diphenyl-^13^C_2_-acetylene	CD_3_CN	PE (500–600 *μ*m)

The results of a pulse calibration obtained using the pulse sequence in Figure 4A and with B_0_ inhomogeneities compensation obtained by supplying a 3.2 A current to the Z-shim coil is reported in Figure 4B. Note that the signal is null when the low field pulse is an exact 90° pulse because of the high field 90° pulse placed before acquisition. In this experiment, the transport time *τ*_*tr*_ was set to 4 s (much shorter than the sample ${\text{T}}_{1}^{\text{HF}}\left(S1\right)=65\pm$ 4 s). The resulting value of the 90° pulse length is 21.5 *μ*s and is used for all experiments discussed below.

Note also that, because our setup uses radiofrequency pulses in quite a low field, it is important to estimate the size of the Bloch-Siegert shift (Bloch and Siegert, 1940). This shift results from the counter rotating component of the radiofrequency field and it is generally negligible in most high-field NMR conditions. Essentially, the Bloch-Siegert effect contributes with a term proportional to ${\hat{I}}_{z}$ in the spin Hamiltonian. Such term generates an off-resonance effect for the radiofrequency pulse itself, meaning that the pulse rotates the magnetization about an effective axis that is tilted by:

$$\theta_{e}=ArcTan\left(\frac{\omega_{1}}{\omega_{BS}}\right)$$ (1)

with respect to the direction of static magnetic field. In the previous equation, the term *ω*_*BS*_ represents the magnitude of the Bloch-Siegert shift and is calculated as:

$$\omega_{BS}=\frac{\omega_{1}^{2}}{4\omega_{rf}}$$ (2)

with *ω*_1_ being the angular nutation frequency of the applied radiofrequency field and *ω*_*rf*_ its oscillation frequency. In our apparatus, the application of a pulse along the *x*-axis generates a Bloch-Siegert shift which is calculated to be *ω*_*BS*_ = 490.8 Hz. This corresponds to an effective tilt angle of *θ*_*e*_ = 89.6° which is negligibly different from the nominal 90° expected for an *x*-axis pulse.

## Materials and methods

### Sample preparation

Three different samples were used in this paper with the intent to calibrate the new hardware and demonstrate its capabilities.

Sample **S1** was prepared by dissolving 40 mg of ^13^C_1_ sodium pyruvate in 500 *μ*L of D_2_O inside a 5 mm OD Wilmad-Labglass pressure/vacuum valve NMR tube. The molecule was chosen because it gives a single peak and has a long T_1_ of 65 ± 4 s at 7.05 T which minimises signal losses during sample transport time. **S1** is used below to calibrate the low field pulse length and to demonstrate measurements of T_1_, T_2_ and isotropic diffusion in low magnetic field using the new hardware setup operated in field-cycling mode.

For demonstrations involving long-lived spin order, such as the measurement of singlet decay constants, T_S_, or singlet-assisted diffusion NMR experiments, we used the singlet-bearing molecule 1,2-diphenyl-^13^C_2_-acetylene, first introduced by Feng et al. (2013) and synthesised in house according to the novel procedure described below.

Sample **S2** was prepared by dissolving 21.6 mg of 1,2-diphenyl-^13^C_2_-acetylene in 500 *μ*L of acetonitrile-d_3_ inside a 5 mm OD Wilmad-Labglass pressure/vacuum valve NMR tube.

Sample **S3** was prepared by dissolving 90 mg of 1,2-diphenyl-^13^C_2_-acetylene in 500 *μ*L of acetonitrile-d_3_. The solution was poured over polyethylene beads with a diameter distribution of 500–600 *μ*m (purchased from Cospheric CMPS), randomly packed at the bottom of a 10 mm OD Norell High vacuum/pressure tube. The total packing height was 2 cm in order to fully encompass the probe coil region with sufficient excess to ensure that the packing was as uniform as possible across the region of interest. The packing was done by weighing out ca. 0.4 g of the polyethylene beads and adding this to the NMR tube in 2 aliquots. Between each aliquot gentle manual tapping was undertaken and after the addition of all aliquots the sample was manually tapped for 1 min to aid packing. **S3** was intended to serve as a model porous system and is used below to demonstrate the ability of the new hardware to measure the diffusion tensor and other NMR parameters in low magnetic field where the negative effects of susceptibility inhomogeneities, that usually impair those experiments in high field, become negligible. All tubes were modified so that their top valve screws directly into the sample guide rod of the shuttling system. The 10 mm tubes also required modification of the valve to reduce the maximum outer diameter at any point to 9.8 mm as this must pass through the low field probe during shuttling. None of the 3 samples was degassed to remove dissolved oxygen; this is because paramagnetic oxygen dissolved in solution under standard conditions, causes only minor effects on the relaxation times of longitudinal and singlet order in ^13^C-spin pairs. Table 1 resumes all samples used in this work whereas Table 2 resumes the various decay constants measured on these three sample as explained below.

**TABLE 2:** Summary of relaxation decay constant measured in this work. * not supported because not a spin pair, † not measurable because of short T_2_. - not measured.

Sample	${\text{T}}_{1}^{\text{HF}}$ (s)	${\text{T}}_{1}^{\text{LF}}$ (s)	${\text{T}}_{2}^{\text{HF}}$ (s)	${\text{T}}_{2}^{\text{LF}}$ (s)	${\text{T}}_{S}^{\text{HF}}$ (s)	${\text{T}}_{S}^{\text{LF}}$ (s)
**S1**	64 ± 4	70 ± 2	9.5 ± 0.4	16.8 ± 0.6	*	*
**S2**	16 ± 2	13 ± 1	7.0 ± 0.5	-	325 ± 18	261 ± 38
**S3**	21 ± 1	20.5 ± 0.3	0.31 ± 0.04	3.9 ± 0.9	†	268 ± 8

### NMR methods

Measurements of T_1_, T_2_ and T_S_ at high field have been done using the following standard methods: inversion recovery (IR), (Hahn, 1949), carr-purcell-meiboom-gill (CPMG) (Meiboom and Gill, 1958) and magnetisation-to-singlet based methods (M2SS2M), (Pileio et al., 2010), respectively. For measurements in field-cycling mode, ad-hoc pulse sequences were introduced as detailed below. Errors displayed alongside each quantity measured in the experimental section refer to the statistical error from the non-linear regression of the experimental data (the area under the NMR signal acquired in each particular experiments) and are calculated using standard routines in Wolfram Mathematica.

### Chemical synthesis

**FIGURE 5:**
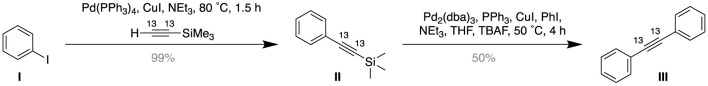
Synthetic route used to prepare 1,2-diphenyl-^13^C_2_-acetylene.

1,2-diphenyl-^13^C_2_-acetylene (**III** in Figure 5) was synthesised via Sonogashira reaction of iodobenzene (**I**) with commercially available trimethyl (phenylethynyl-1,2–^13^C_2_)silane (99 atom % ^13^C, Sigma Aldrich) to give trimethyl (phenylethynyl-1,2–^13^C_2_)silane (**II**), which was subjected to a one-pot desilylation/Sonogashira reaction with idodobenzene, resulting in an overall 49% yield. See Supplementary Material for full procedures, characterisation, and spectral data.

## Results and discussion

### Relaxation of longitudinal order in low magnetic field

**FIGURE 6:**
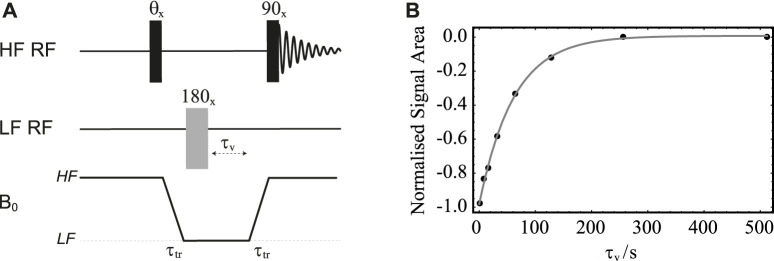
**(A)** Pulse sequence in field-cycling mode used to measure T_1_ in LF. **(B)** Normalised signal area under the peak in the ^13^C-NMR spectrum of sample **S1** acquired in HF plotted against the variable time *τ*_v_. Solid circles are experimental measurements whereas the grey line is the best fit to $s_{{\text{T}}_{1}^{\text{LF}}}$ .

Low-field measurements of the decay constant of longitudinal order in low field ( ${\text{T}}_{1}^{\text{LF}}$ ) were done using the field-cycling version of the inversion recovery pulse sequence shown in Figure 6A. The flip angle of the initial high-field pulse, *θ*, is cycled between 0° and 180° in two successive transients while the receiver phase alternates between 0° and 180° to compensate for magnetization build up during sample transport. The longitudinal magnetization prepared in the HF probe, inverted or not by the initial *θ* pulse, is then transferred to LF where a 180° radiofrequency pulse is applied. After a variable time *τ*_v_, the sample is shuttled back to HF where a 90° pulse generates transverse magnetization that is detected in the HF probe. The experiment is repeated for incremental values of the variable time interval *τ*_v_ and ${\text{T}}_{1}^{\text{LF}}$ is retrieved by fitting the normalised signal area plotted *versus* *τ*_v_ to the function: $s_{{\text{T}}_{1}^{\text{LF}}}=A+Be^{-\tau_{\text{v}}/{\text{T}}_{1}^{\text{LF}}}$ , as in conventional IR experiments.

The procedure is demonstrated with the use of sample **S1** and results are plotted in Figure 6B. In these experiments, the sample is polarised in the HF probe for 180 s and shuttled between HF and LF probes in 4 s. The variable time *τ*_v_ is incremented between 1 and 512 s in 8 steps. The normalised signal area of the sample’s ^13^C-NMR spectrum acquired in HF is plotted against *τ*_v_ in Figure 6B. The experimental points are fitted to $s_{{\text{T}}_{1}^{\text{LF}}}\left(\tau_{\text{v}}\right)$ and yielded ${\text{T}}_{1}^{\text{LF}}\left(S1\right)=70\pm 2$ s, which is quite close to the value of ${\text{T}}_{1}^{\text{HF}}$ (**S1**) measured at 7.05 T on the same sample.

### Relaxation of transverse order in low magnetic field

**FIGURE 7:**
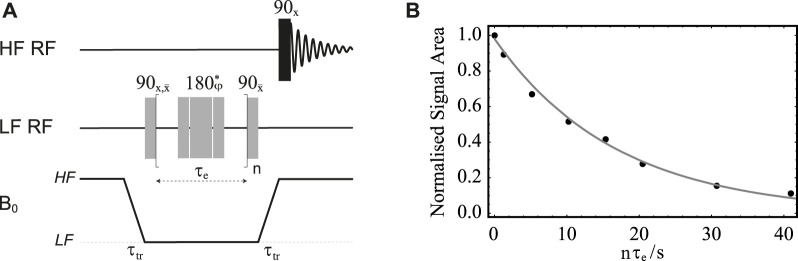
**(A)** Pulse sequence in field-cycling mode used for measurement of T_2_ in LF. The central 180° pulse marked with an asterisk is a composite pulse implemented as 90_*x*_180_*y*_90_*x*_; its overall phase is cycled as $\varphi =\left\{x,x,\bar{x},\bar{x},\bar{x},x,x,\bar{x},\bar{x},\bar{x},x,x,x,\bar{x},\bar{x},x\right\}$ through the *n* repetitions. **(B)** Normalised signal area under the peak in the ^13^C-NMR spectrum of sample **S1** acquired in HF plotted against *nτ*_e_. Solid circles are experimental measurements whereas the grey line is the best fit to $s_{{\text{T}}_{2}^{\text{LF}}}$ .

The low-field value of the decay constant of transverse order ( ${\text{T}}_{2}^{\text{LF}}$ ) is measured using the field-cycling version of the carr-purcell-meiboom-gill pulse sequence shown in Figure 7A. The phase of the initial low field pulse is cycled between 0° and 180° while the receiver phase is alternated between 0° and 180° in two successive transients to compensate for magnetization build up during sample transport. The central 180° pulse is a composite pulse implemented as 90_*x*_180_*y*_90_*x*_ with its overall phase cycled as $\varphi =\left\{x,x,\bar{x},\bar{x},\bar{x},x,x,\bar{x},\bar{x},\bar{x},x,x,x,\bar{x},\bar{x},x\right\}$ through the *n* echo repetitions. To measure ${\text{T}}_{2}^{\text{LF}}$ , the echo time *τ*_e_ is fixed to a small value (10 ms in our case) so to minimise diffusion-induced signal attenuation and the echo block is repeated for a variable number of times, *n*. After the echo train, transverse magnetization is stored along the field *z*-direction via a 90° pulse applied in LF. The sample is shuttled back to HF where a signal is detected after the application of a 90° pulse in the HF probe. The normalised signal area of the signal acquired in HF is plotted against *n* × *τ*_e_ and the curve is fitted to the exponential decay: $s_{{\text{T}}_{2}^{\text{LF}}}\left(n\tau_{\text{e}}\right)=Ae^{-n\tau_{\text{e}}/{\text{T}}_{2}^{\text{LF}}}$ , as in conventional CPMG experiments.

The procedure is demonstrated with the use of sample **S1** and results are reported in Figure 7B. In these experiments, the sample is firstly polarised in the HF probe for 180 s. The echo time, *τ*_e_, is fixed to 20 ms and the echo block repeated *n* times, with *n* incremented between 1 and 2048 in 8 steps. The sample transport time is set to 4 s. The plot of the normalised signal area acquired in HF *versus* *nτ*_e_ is reported in Figure 7B. The experimental points are fitted to $s_{{\text{T}}_{2}^{\text{LF}}}$ and yielded a value of ${\text{T}}_{2}^{\text{LF}}\left(S1\right)=16.8\pm 0.6$ s. Incidentally, the transverse relaxation decay constant for the same sample in high field was found to be ${\text{T}}_{2}^{\text{HF}}\left(S1\right)=9.6\pm 0.4$ s.

### Isotropic diffusion in low magnetic field

**FIGURE 8:**
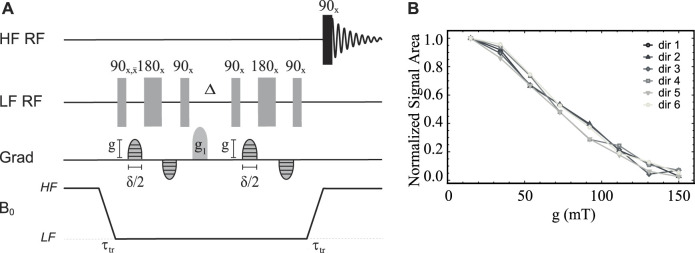
**(A)** Pulse sequence in field-cycling mode used for measurement of diffusion in LF. **(B)** Normalised signal area under the peak in the ^13^C-NMR spectrum of sample **S1** acquired in HF plotted against the gradient strength *g*. Solid lines are added as a guide for the eyes.

To measure molecular diffusion in low field, we have introduced the field-cycling version of the stimulated-echo pulse sequence shown in Figure 8A. The phase of the initial low field pulse is cycled between 0° and 180° while the receiver’s phase is alternated between 0° and 180° in two successive transients to compensate for magnetization build up during sample transport. To measure the isotropic diffusion coefficient D_0_, one would typically fix the value of the diffusion time Δ and vary the strength of the bipolar gradient *g*, usually expressed as a percentage of the maximum gradient available. The gradient duration *δ* is set to be much shorter than Δ and the actual values of Δ and *δ* are chosen such that the signal decays nicely while *g* is varied within a suitable interval of the available maximum gradient strength. The gradient *g*_1_ is a spoiler gradient to clean up the signal from unwanted byproducts. The normalised signal area plotted *versus* *g* is then fitted to the diffusion curve: $s_{{\text{D}}_{0}}=Ae^{-{\text{D}}_{0}{\left(\gamma \delta g\right)}^{2}\left(\Delta -\delta /3\right)}$ , as in conventional diffusion experiments (Stejskal and Tanner, 1965). If the whole diffusion tensor is required, as in diffusion tensor imaging (DTI) experiments, then the procedure above is repeated for a minimum of 6 times, each time choosing a different direction along which the pulsed field gradient is applied. This is because the diffusion tensor is a symmetric rank-2 tensor with six independent values. The choice of these six directions can be optimised once for all and several optimized set of directions are available in literature. We have chosen to work with the 6 directions obtained via a repulsion algorithm (Jones et al., 1999). The results of the 6 experiments, each containing a number of experimental points resulting from the increment of the value of *g* along each chosen direction, are then processed together to yield the six independent values of the diffusion tensor following standard procedures (Mori and Toumier, 2013).

Diffusion NMR experiments in low magnetic field are complicated by the effects of concomitant (a.k.a. Maxwell) gradients (Baron et al., 2012). Basically, in order to satisfy Maxwell’s laws, magnetic field gradients must have null divergence and curl. Gradient system used in NMR and MRI generally have cylindrical symmetry with coils built to generate a magnetic field pulse (here assumed uniform) along the static magnetic field direction, and whose strength varies along some direction in space. However, according to Maxwell equations, those coils generate the following field vectors (Bernstein et al., 1998; Meier et al., 2008):

$$\begin{array}{cc} {\vec{B}}_{G_{x}} & =\left\{G_{x}z,\;0,\;G_{x}x\right\}\\ {\vec{B}}_{G_{y}} & =\left\{0,\;G_{y}z,\;G_{y}y\right\}\\ {\vec{B}}_{G_{z}} & =\left\{-\frac{G_{z}}{2}x,\;-\frac{G_{z}}{2}y,\;G_{z}z\right\} \end{array}$$ (3)

with

$$G_{x}=\frac{\partial B_{z}}{\partial x};\;G_{y}=\frac{\partial B_{z}}{\partial y};\;G_{z}=\frac{\partial B_{z}}{\partial z};$$ (4)

This means that by turning on a gradient along x with the intention of producing a magnetic field component that points along z but whose strength varies along the *x*-axis, one necessarily produces magnetic field components pointing along the *x* and *y* directions as well. These unwanted components are known as concomitant or Maxwell gradients. Taking into account the underlying static magnetic field ${\vec{B}}_{S}=\left\{0,0,B_{0}\right\}$ , the magnitude of the total field when in presence of a gradient pulse with these characteristics, expanded in a Taylor series truncated to the second order in the gradient strength, is:

$$\begin{array}{cc} \left\|{\vec{B}}_{G_{x}}\right\| & =\sqrt{\left({\vec{B}}_{S}+{\vec{B}}_{G_{x}}\right)\cdot \left({\vec{B}}_{S}+{\vec{B}}_{G_{x}}\right)}\approx B_{0}+G_{x}x+\frac{G_{x}^{2}z^{2}}{2B_{0}}\\ \left\|{\vec{B}}_{G_{y}}\right\| & =\sqrt{\left({\vec{B}}_{S}+{\vec{B}}_{G_{y}}\right)\cdot \left({\vec{B}}_{S}+{\vec{B}}_{G_{y}}\right)}\approx B_{0}+G_{y}y+\frac{G_{y}^{2}z^{2}}{2B_{0}}\\ \left\|{\vec{B}}_{G_{z}}\right\| & =\sqrt{\left({\vec{B}}_{S}+{\vec{B}}_{G_{z}}\right)\cdot \left({\vec{B}}_{S}+{\vec{B}}_{G_{z}}\right)}\approx B_{0}+G_{z}z+\frac{G_{z}^{2}\left(x^{2}+y^{2}\right)}{8B_{0}} \end{array}$$ (5)

Hence, in the presence of a field gradient applied along the *z* direction, the actual magnetic field experienced by a certain molecule in a given position within the sample (to the second order) points slightly off the *z*-axis as dictated by the third line of Eq. 3. Moreover, its magnitude will also contain the undesired terms proportional to *×* ^2^ and *y* ^2^ as dictated by the third line of Eq. 5. Similar unwanted tilting of the local magnetic field direction and extra terms in its magnitude are present in case of gradients applied along the *x* and *y* directions. The extent of the undesired term is proportional to the ratio between the gradient strength and the static magnetic field. In conventional high field experiments, where the static magnetic field is of the order of a few Tesla and the maximum available gradient strengths are of the order of a Tesla per meter, the effect of Maxwell gradients are negligible. In our current setup, instead, the field gradients are applied in a magnetic field of 46.4 mT and the maximum gradient strength available is 1.5 T m^−1^. Assuming the NMR sample is 20 mm long and centred in the LF sweet spot, the field experienced by spins located 10 mm above the LF sweet spot is calculated to be 62.4 mT when the gradient is applied at full strength. In those circumstances, the total magnetic field is tilted by about 10° away from the *z*-axis. To mitigate these phenomena the maximum gradient strength used in LF must be limited. The same calculations above redone for a maximum gradient strength of 75 mT m^−1^ (5% of the maximum available in our hardware) would result in a field intensity of 47.1 mT for spins located 10 mm above the LF sweet spot and the tilt angle away from the *z*-axis would only be 0.6°. In order to perform NMR diffusion experiments with gradients ranging from 0 to a maximum of 75 mT m^−1^ one has to increase either (or both) the diffusion time (Δ in Figure 8A) or the gradient pulse duration (*δ* in Figure 8A). The relaxation time of longitudinal spin order, T_1_, sets a limit to the maximum useable value of Δ while the value of *δ* must be kept much smaller than Δ to fulfill the approximations which underpins the theoretical description of diffusion-NMR experiments (Callaghan, 2011).

Fortunately, in singlet assisted diffusion NMR, Δ can be of the order of many tens of seconds while *δ* can remain in the milliseconds regime. Hence, gradient strengths of a few mT m^−1^ can provide meaningful diffusion information despite the presence of concomitant gradients. This is yet another important feature of long-lived spin order and singlet-assisted diffusion NMR.

Before introducing singlet-assisted diffusion NMR experiments, though, we demonstrate how the new hardware can be used to measure the diffusion tensor with the use of the pulse sequence in Figure 8A and sample **S1**. For this experiment, the sample was polarised in HF for 180 s and then transported in LF with a transport time of 4 s. The diffusion time Δ was set to 300 ms and the duration of the diffusion gradient, *δ* was set to 20 ms. The gradient *g*_1_ had a duration of 2 ms and a strength of 0.26 T m^−1^ and was applied along the negative *z* direction. The diffusion gradient *g* was varied from 15 to 150 mT m^−1^ (1%–10% of the available maximum) in 8 linearly spaced values. The whole experiment was repeated 6 times with the gradient *g* each time applied along a different direction (see Table 3), optimised using a repulsion algorithm in Jones et al., (1999).

**TABLE 3:** The x, y and z component (*d*_*x*_, *d*_*y*_, *d*_*z*_) of the unitary vectors pointing along the six direction of space optimised according to a repulsion algorithm in Jones et al., (1999) and used for diffusion tensor imaging experiments in this paper.

	dir 1	dir 2	dir 3	dir 4	dir 5	dir 6
*d*_*x*_	1.000	0.447	0.447	0.447	0.447	−0.447
*d*_*y*_	0.000	0.895	0.277	−0.724	−0.724	−0.277
*d*_*z*_	0.000	0.000	0.850	−0.525	0.525	0.850

The normalised signal area of sample **S1**’s ^13^C-NMR spectrum acquired in HF is plotted against *g* for all six directions in Figure 8B.

As expected in the case of isotropic diffusion, all six directions give curves that are identical within error. This is because in isotropic solutions unrestricted molecular diffusion is the same in any direction of space. Furthermore, the 48 experimental points, 8 per direction, were fitted together to yield a nearly spherical diffusion tensor with a fractional anisotropy FA(**S1**) = 0.07 ± 0.02 and an isotropic diffusion coefficient D_0_(**S1**) = (6.4 ± 0.2) × 10^–10^ m s^−2^. The small but not exactly zero value of fractional anisotropy is likely due to small differences in the gradient performances along the different directions, which is typically observed in DTI experiments and can be calibrated for. The measured value of the diffusion coefficient falls in the expected range for a small molecule in deuterated water solutions. When the same experiment is repeated using Δ = 150 ms, *δ* = 2 ms and *g* varied from 15 to 1,500 mT m^−1^ (1%–100% of the available maximum) the resulting diffusion curves cannot be fitted to the diffusion curve because of the effect of concomitant gradients (not shown).

### Relaxation of singlet order in porous systems

As briefly explained in the introduction, measuring the lifetime of singlet spin order in a sample containing a porous matrix in high magnetic fields is complicated, and often made impossible, by the mismatch between the magnetic susceptibility of the material constituting the porous structure and the one of the imbibed liquid. These susceptibility inhomogeneities, even if just of the order of a few part-per-millions, generates a T_2_-like mechanism that relaxes transverse magnetisation in milliseconds (Cartlidge et al., 2022) and leaves no time to generate singlet spin order because this typically requires hundreds’ of milliseconds (Carravetta et al., 2004; Carravetta and Levitt, 2004; DeVience et al., 2013; Kiryutin et al., 2013; Theis et al., 2014; Pileio, 2020). Our group is particularly interested in exploiting the extended lifetime of singlet order to measure the diffusion tensors and tortuosity in porous media. For this, access to the long-lived singlet states of a “spy” molecule imbibed within the pores of the medium is fundamental. Fortunately, the relaxation phenomena due to spin diffusing in porous media are field dependent and become negligible in low magnetic field. This very fact is what has driven the development of the dual-core equipment described in this paper.

As a first demonstration of the new opportunities offered by our hardware, we measured the low field value of the decay constant of singlet spin order ( ${\text{T}}_{\text{S}}^{\text{LF}}$ ) in a sample where singlet-bearing molecules diffuse between the pores of a random packing of spherical beads (sample **S3**). Long-lived singlet order can be created in virtually any two-spin-1/2 system where the two spins are inequivalent. However, it is much more convenient to work with nearly-equivalent spin pairs because in such a case the singlet order is almost an eigenstate of the spin Hamiltonian and therefore its does not need to be sustained by transport in low-field (Carravetta et al., 2004) or radiofrequency irradiation (Carravetta and Levitt, 2004). Spin inequivalence can be of chemical or magnetic nature. In chemically inequivalent spin pairs, we have a difference in chemical shift between the two spins; to have a nearly-chemically-equivalent spin pair we need the difference in chemical shift frequency between the two nuclei (|*ω*_1_ − *ω*_2_|) to be much smaller than their mutual scalar coupling (*J*_12_), i.e., |*ω*_1_ − *ω*_2_|≪ *J*_12_. In magnetically inequivalent pairs, we have a different scalar coupling between the two spins and a remote third spin in the molecule; to have a nearly-magnetically-equivalent spin pair, we need the difference in the scalar coupling between each spin in the pair and the third nucleus (|*J*_13_-*J*_23_|) to be much smaller than their mutual scalar coupling (*J*_12_), i.e., |*J*_13_ − *J*_23_|≪ *J*_12_. In the context of this work, it is more convenient to work with nearly-magnetically-equivalent spin pair. This is because the parameters to access the singlet order will not change between HF and LF. Conversely, nearly-chemical-equivalence is field dependent because this relies on the chemical shift frequency. The two ^13^C labels introduced in compound **III** (see Figure 5) constitute a nearly-magnetically-equivalent spin pair through their small scalar coupling to the protons in ortho on the ring. The scalar coupling between the two ^13^C nuclei is $J_{C_{1}C_{2}}=182$ Hz, whereas the scalar coupling between the ^13^C nuclei and the protons in ortho are $J_{C_{1}H_{o}}=5.5$ Hz and $J_{C_{2}H_{o}}=-0.6$ Hz. (Feng et al., 2013). The scalar couplings with the protons in meta and para are very small and can be effectively neglected.

**FIGURE 9:**
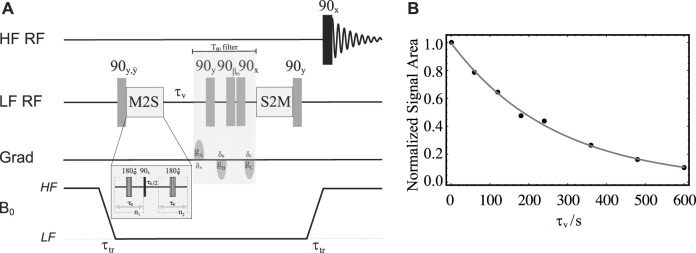
**(A)** Pulse sequence in field-cycling mode used for measurement of T_S_ in LF. **(B)** Solid circles are experimental normalised signal areas acquired in HF and plotted against the variable time *τ*_*v*_. The solid gray line is the best fit to $s_{{\text{T}}_{\text{S}}}$ .

To calibrate the parameters to access singlet order and to have a reference value for the singlet order decay constant in a non-porous system, we have first conducted experiments using sample **S2**. For the actual measurement of ${\text{T}}_{\text{S}}^{\text{LF}}$ we have produced a version of the M2SS2M pulse sequence adapted to work in field-cycling mode on our equipment. A sketch of the pulse sequence is reported in Figure 9A. The sample is first polarised in HF and then shuttled to LF where a first 90° pulse creates transverse polarization. The phase of this pulse in cycled between 90° and 270° while the receiver phase is also cycled between 90° and 270° across two successive transients so to compensate for polarization buildup during both *τ*_*v*_ and transport. The M2S block has been described in detail elsewhere (Pileio et al., 2010). It converts transverse magnetisation into singlet order through a train of spin echoes synchronised with the spin system’s parameters. To maximise efficiency, theory predicts that the echo time must be set to $\tau_{e}=1/\left(4{\left(J_{12}^{2}+{\left(J_{13}-J_{23}\right)}^{2}\right)}^{1/2}\right)$ and the number of echoes to *n*_1_ = *π*/(2 *ArcTan*(*J*_13_ − *J*_23_/*J*_12_)) with *n*_2_ = *n*_1_/2 (Feng et al., 2013). The central 180° pulse in the M2S block is a composite pulse implemented as 90_*x*_180_*y*_90_*x*_ with its overall phase cycled as $\varphi =\left\{x,x,\bar{x},\bar{x},\bar{x},x,x,\bar{x},\bar{x},\bar{x},x,x,x,\bar{x},\bar{x},x\right\}$ through the *n*_1_ and *n*_2_ repetitions. After a variable time, *τ*_*v*_ (incremented in a series of experiments to measure the singlet order decay constant), a *T*_00_ filter is used to filter out unwanted byproducts (Pileio, 2020). The S2M block that follows is the time reverse of the M2S and converts singlet order back to transverse magnetization. This latter is stored along the static magnetic field direction by the last 90° pulse in LF. The sample is then transported back to HF for a 90° pulse and successive signal detection.

To calibrate the value of *τ*_*e*_ and *n*_1_ required by the pulse sequence in Figure 9A, we run a train of synchronised spin echoes where we varied *τ*_*e*_ and *n*_1_, in turn, and around the theoretical values obtained using the equations above and the experimental values of the scalar couplings involved therein. Note that since the magnetic in-equivalence does not depends on the value of the static magnetic field, these optimisations can be done either in HF or in LF. This calibration, run on sample **S2** in HF, returned the following values: *τ*_*e*_ = 2.75 ms, *n*_1_ = 48 (and *n*_2_ = *n*_1_/2 = 24). These values were then used to measure a ${\text{T}}_{\text{S}}^{\text{LF}}\left(S2\right)=261\pm 38$ s. In these experiments, the transport time was fixed to 3 s and the variable time *τ*_*v*_ was incremented from 1 to 600 s in 8 steps. The value of ${\text{T}}_{\text{S}}^{\text{LF}}$ we found is consistent with the one reported by Feng et al. (2013) for the same molecule (there measured at a different field and in a different solvent). Incidentally, we measured a value of ${\text{T}}_{1}^{\text{LF}}$ (**S2**) = 13 ± 1 s for the same sample and using the pulse sequence in Figure 6A. The longitudinal order decay constant for the same sample but measured in high field was ${\text{T}}_{1}^{\text{HF}}$ (**S2**) = 16 ± 2 s. The singlet order dacay constant in high field was ${\text{T}}_{\text{S}}^{\text{HF}}\left(S2\right)=325\pm 18$ s.

Successively, using the same pulse sequence in Figure 9A and the values of *τ*_*e*_ and *n*_1_ taken from the optimization above, the value of ${\text{T}}_{\text{S}}^{\text{LF}}$ was measured for sample **S3** as a model for porous media applications. For this experiment, the transport time was fixed to 3 s and the variable time *τ*_*v*_ was incremented from 1 to 600 s in 8 steps. The resulting experimental normalised signal areas are shown as solid circles in Figure 9B. These were fitted to the exponential decay curve $s_{{\text{T}}_{\text{S}}^{\text{LF}}}=Ae^{-\tau_{v}/{\text{T}}_{\text{S}}^{\text{LF}}}$ to yield a singlet decay time constant of ${\text{T}}_{\text{S}}^{\text{LF}}$ (**S3**) = 268 ± 8 s, which is consistent, within errors, with the value earlier found for sample **S2**, thus confirming that the singlet order decay constant is unaffected by the presence of the beads. Note that the smaller error in the measurement of ${\text{T}}_{\text{S}}^{\text{LF}}$ for **S3** (compared to what obtained for **S2**) may be due to the presence, in **S3**, of the beads that stop thermal convection. Also note that such an experiment cannot be performed in HF since the susceptibility difference of 2.8 ppm, between the PE beads and the acetonitrile solution, is responsible for a very short T_2_ in HF, ${\text{T}}_{2}^{\text{HF}}$ (**S3**) = 0.31 ± 0.04 s. Incidentally, we measured a value of ${\text{T}}_{1}^{\text{LF}}$ (**S3**) = 20.5 ± 0.3 s for the same sample.

### Singlet-assisted diffusion NMR in porous media

As a final example, we have addressed the problem of measuring diffusion in porous media and in the long-time regime, i.e., when the diffusion time is long enough so that molecules can travel, on average, for much longer distances than the average pore size. These experiments provide important information such as structural anisotropy, shape and orientation of cavities and channels, as well as tortuosity, i.e., a measure of how difficult is for a diffusing molecule to cover a certain distance as it moves across the pores of a porous structure.

**FIGURE 10:**
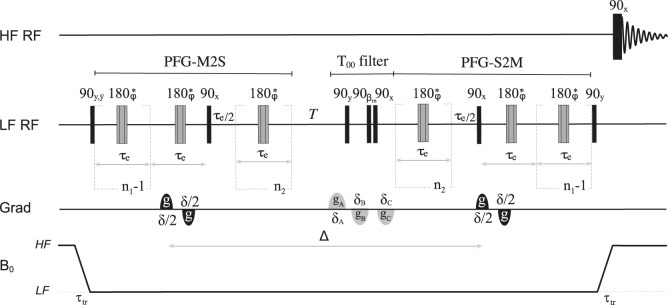
Pulse sequence used for singlet-assisted diffusion experiments in a field-cycling mode.

The pulse sequence introduced to measure the diffusion of singlet-bearing molecules imbibed in porous media is shown in Figure 10. It is a version of the singlet-assisted-diffusion NMR sequence presented in Tourell et al. (2018), here adapted to work in field-cycling mode. Recalling the discussion above regarding the pulse sequence in Figure 9A, and in analogy with the more conventional stimulated echo sequence (STE), the pulse sequence in Figure 10 marks molecular positions with a bipolar gradient placed during the last echo of the *n*_1_ train of the M2S block (now labelled as PFG-M2S to outline the presence of the pulsed field gradient). After storage of the magnetisation as long-lived spin order, molecular positions are decoded through the use of an additional bipolar pulsed field gradient placed during the first echo of the *n*_1_ train of the S2M block. The distance between the two bipolar gradients is the diffusion time, Δ. Because molecular position is stored as long-lived singlet order, the diffusion time is amenable to be minutes long rather than the few seconds allowed by longitudinal order exploited in STE experiments.

As for all other pulse sequences introduced in this paper, magnetization is stored along the static magnetic field direction before transport to HF, and detection is achieved following a 90° pulse in the HF probe. To measure diffusion along a given direction, the bipolar pulses are applied along the chosen direction. To retrieve the whole diffusion tensor, the same experiment is repeated with bipolar pulsed field gradients applied along a minimum of 6 independent directions. Finally, to measure tortuosity along a certain direction of space, the diffusion coefficient is measured as a function of the diffusion time Δ to obtain *D*(Δ). The ratio *D*(Δ)/*D*_0_ tends to the tortuosity value as Δ tends to infinity (Latour et al., 1993). In practice, an asymptotic value of *D*(Δ)/*D*_0_ is reached once the diffusion time is long enough for molecules to travel across many pores and probe a representative part of the structure.

**FIGURE 11:**
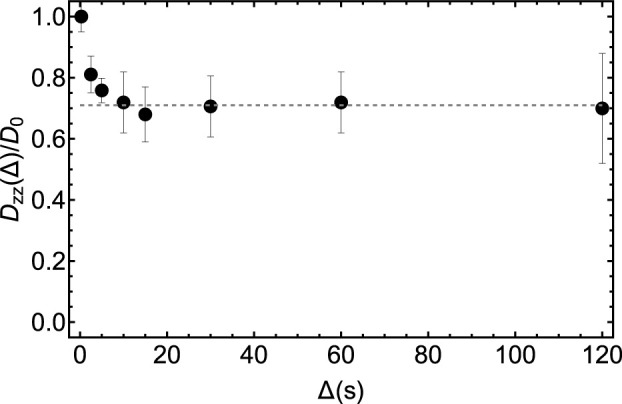
Experimental measurements of the diffusion coefficient along the *z*-direction in the LF probe obtained by using the pulse sequence in Figure 10 and for different values of the diffusion time, Δ. The dashed gray line has been added to guide the eyes towards the asymptotic value of the experimental tortuosity.

In Figure 11 we report the result of a set of low-field measurements of the ratio *D*(Δ)/*D*_0_ for sample **S3** and for several values of the diffusion time. In these experiments, we set *τ*_*e*_ = 2.75 ms and *n*_1_ = 48 from the optimization above. The transport time was fixed to 3 s. The duration of the bipolar gradient, *δ*, was set to 2 ms while Δ was varied from 2.5 to 120 s as shown in Figure 11. For each value of Δ, the gradient strength of the bipolar gradients, *g*, was varied in 4 steps and within a range that is different for different values of Δ, and chosen so to have a good sampling of the diffusion equation while keeping the maximum strength low enough to avoid the above discussed complications due to concomitant gradients. Namely, *g* was ranging within the interval 1%–15% for Δ = 2.5 s and within the interval 1%–3% for Δ = 120 s, with the percentage figure referring to the percentage of the maximum available gradient (1.5 T m^−1^). The bipolar gradients were applied along the *z*-direction. As expected, the ratio *D*_*zz*_(Δ)/*D*_0_, plotted in Figure 11, reaches an asymptotic value as Δ is increased. Such limiting value corresponds to the tortuosity of the system. Depending on the porosity and the looseness of the packing, tortuosity varies in randomly-packed-bead systems; simulations done on systems with porosity between 0.36 and 0.46 and for various methods of packings, give a tortuosity value that varies within 0.71–0.76 (Khirevich et al., 2011). Our experimental value of 0.71 (dotted line in Figure 11) falls in the right interval, although we have not properly characterised our packing because this is beyond the scope of this paper.

Finally, we have used the pulse sequence in Figure 10 to measure the full diffusion tensor in LF and for two different values of Δ, namely, 2 s and 30 s. For these experiments we have used the parameters: *τ*_*e*_ = 2.75 ms, *n*_1_ = 48, *n*_2_ = 24, *τ*_*tr*_ = 3 s, *δ* = 2 ms. The gradient strength, *g* was varied in 4 steps between 1% and 15% of the maximum for Δ = 2 s and between 1% and 6% for Δ = 30 s. For each value of Δ, the bipolar gradients were applied, in successive experiments, along the six different directions of space reported in Table 3. The 24 (six times four) points were fitted together to reconstruct the whole diffusion tensor according to established procedures (Basser et al., 1994; Mori and Toumier, 2013). The tensor was then diagonalised to obtain the diffusion coefficient along the three principal direction of diffusion. The diagonalised experimental diffusion tensors so derived are:

$$D^{\prime }\left(S3,2\text{ s}\right)=\left(\begin{array}{ccc} 1.1\pm 0.3 & 0 & 0\\ 0 & 1.2\pm 0.1 & 0\\ 0 & 0 & 1.5\pm 0.2 \end{array}\right)\times 10^{-9}{\text{m}}^{2}\;{\text{s}}^{-1}$$ (6)

with an associated fractional anisotropy FA(**S3**) = 0.14 and:

$$D^{\prime }\left(S3,30\text{ s}\right)=\left(\begin{array}{ccc} 0.70\pm 0.08 & 0 & 0\\ 0 & 0.96\pm 0.01 & 0\\ 0 & 0 & 1.3\pm 0.2 \end{array}\right)\times 10^{-9}{\text{m}}^{2}\;{\text{s}}^{-1}$$ (7)

with an associated fractional anisotropy FA(**S3**) = 0.30. As expected, the value of FA for the structure becomes more apparent when the experiment is done at larger Δ. By taking the ratios between the diffusion coefficients along the same principal direction but for different value of Δ, namely, ${\text{D}}_{\alpha \alpha }^{\prime }$ (30 s)/ ${\text{D}}_{\alpha \alpha }^{\prime }$ (2 s), one gets 0.64, 0.80 and 0.87 for the principal x-, y- and z-directions, respectively. This highlights how tortuosity is different along different directions of space and how this information can be retrieved by measuring the full diffusion tensor as a function of the diffusion time.

## Conclusion

In this paper, we have presented a new hardware development consisting in a dual-core NMR spectrometer with high-resolution detection facility in high field (7.05 T) and both radiofrequency and 3-axis gradient facilities in low field (46.4 mT). The hardware is complemented by a sample shuttle with precision 3-axis positioning to work in field cycling mode. The equipment is fully controlled by the spectrometer console through the pulse programs and it has been mainly developed to perform diffusion experiments in porous materials. These kind of experiments in porous media are usually impaired (and often invalidated) by the short decay time of the transverse spin magnetization resulting from magnetic susceptibility inhomogeneities between the medium and the imbibed solution. Because such effect is field dependent, the ability to use RF and field pulse gradients in low field, while retaining the high field facility for sample polarisation and detection, makes those experiments now doable in our apparatus. After presenting the hardware, we have discussed a series of calibration procedures and examples of experiments that can be performed on it, namely, measurement of T_1_ and T_2_ relaxation decay constants, as well as diffusion in one or multiple directions. Moreover, we showed how this new hardware gives access to manipulations of long-lived spin order in porous media (previously impossible where susceptibility inhomogeneities are larger than a few ppm’s) by measuring the relaxation decay constant of singlet spin order in a test sample where molecules are diffusing within randomly-packed plastic beads. Additionally, we demonstrated how the new hardware gives unprecedented access to tortuosity and whole diffusion tensors in porous media. This information is of relevance in several disciplines such as material science and biology. For example, we plan to use this new hardware to measure diffusion and tortuosity within the gas-diffusion-layer of fuel cells. Such experimental data are relevant, for example, for simulations of fuel cells functionalities. We are also working on using this new hardware to measure diffusion and tortuosity in biological tissues obtained by growing cells on 3D-printed scaffoldings. Information on those systems are relevant to the field of tissue engineering either in tissue regeneration applications or for the characterisation of 3D models of cancer.

## Data Availability

The original contributions presented in the study are included in the article/Supplementary Material, further inquiries can be directed to the corresponding author.
