# Supplementary material for: A dual-core NMR system for field-cycling singlet assisted diffusion NMR
Source: Front Chem. 2023 Jul 5;11:1229586. doi: 10.3389/fchem.2023.1229586 (PMC10354561; doi:10.3389/fchem.2023.1229586)
Supplement: Supplementary file 1 [file DataSheet1.docx]

**Supplementary Material**

**A dual-core NMR system for Field-Cycling
Singlet Assisted Diffusion NMR**

Thomas B. R. Robertson,† Rose C. Bannister,† Topaz A. A. Cartlidge,† Thimo Hugger,‡ Sebastien Breham,‡ Klaus Zick,‡ Frank Engelke,‡ Sam Thompson,† and Giuseppe Pileio∗†

*†School of Chemistry, University of Southampton, United Kingdom SO17 1BJ
‡Bruker Biospin GmbH, Silberstreifen, D-76287 Rheinstetten, Germany
*E-mail: g.pileio@soton.ac.uk*

# Table of contents

Contents

[Table of contents 2](#_Toc138180939)

[Low Field Probe 3](#_Toc138180940)

[Hardware Construction 3](#_Toc138180941)

[Low field probe power simulations 7](#_Toc138180942)

[Probe Requirements 7](#_Toc138180943)

[Reference Power Calculations 7](#_Toc138180944)

[Low field Temperature 13](#_Toc138180945)

[Probe Temperature Limit Determination 13](#_Toc138180946)

[Effect of Shim coil Heating on Sample Temperature 15](#_Toc138180947)

[Shim Measurements 18](#_Toc138180948)

[Chemical Synthesis 18](#_Toc138180949)

[General information 18](#_Toc138180950)

[Solvents and reagents 18](#_Toc138180951)

[Chromatography and equipment 18](#_Toc138180952)

[Analytical techniques 18](#_Toc138180953)

[Synthetic Scheme 19](#_Toc138180954)

[Trimethyl(phenylethynyl-1,2-^13^*C*_2_)silane **II** 19](#_Toc138180955)

[1,2-Diphenylethyne-1,2-^13^*C*_2_ **III** 20](#_Toc138180956)

[Spectra 21](#_Toc138180957)

[Single Echo Simulations 27](#_Toc138180958)

[Sample Spectra Comparison 28](#_Toc138180959)

[References 29](#_Toc138180960)

# Low Field Probe

## Hardware Construction

The low field (LF) probe which is a central part of the equipment was designed and manufactured in collaboration with Bruker BioSpin GmbH and is described in the main text. The below section shows a schematic view of this probe and the associated components assembled into the hardware described

herein.


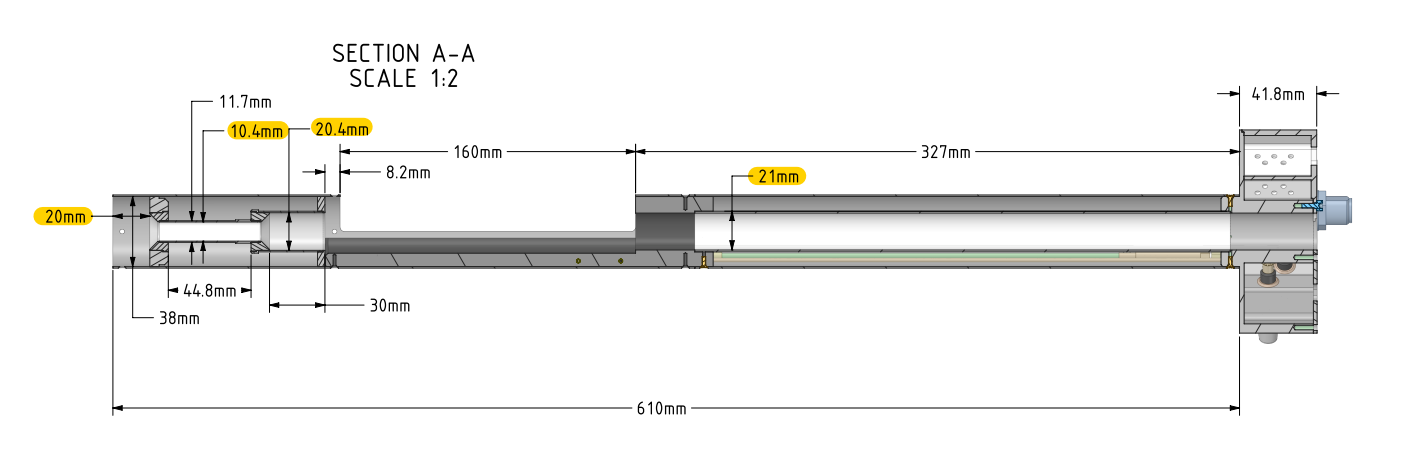


Figure S1. Schematic of probe assembly including base with interior free space highlighted in yellow.

Within the interior of the probe an insert was fitted to allow the smooth motion of a sample transport rod of 7 mm square section ASA, this insert, and all its parts are shown in Figures S2 to S4.


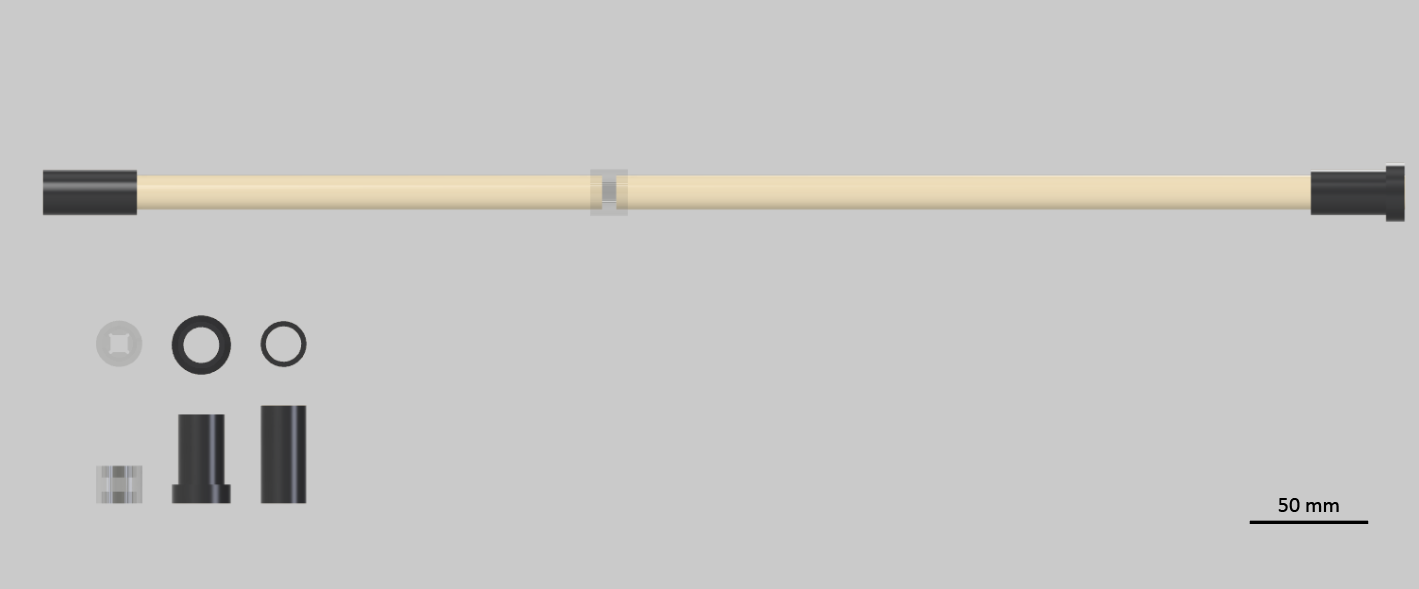
Figure S2. (A) Low field probe insert as appear when fully assembled; (B) polycarbonate guide insert with square section 7.1 mm hole to ensure sample remains aligned within the xy plane; (C) nylon component to centre insert at top of probe body; (D) nylon component to centre insert just above the probe coil.

A

B

C

D


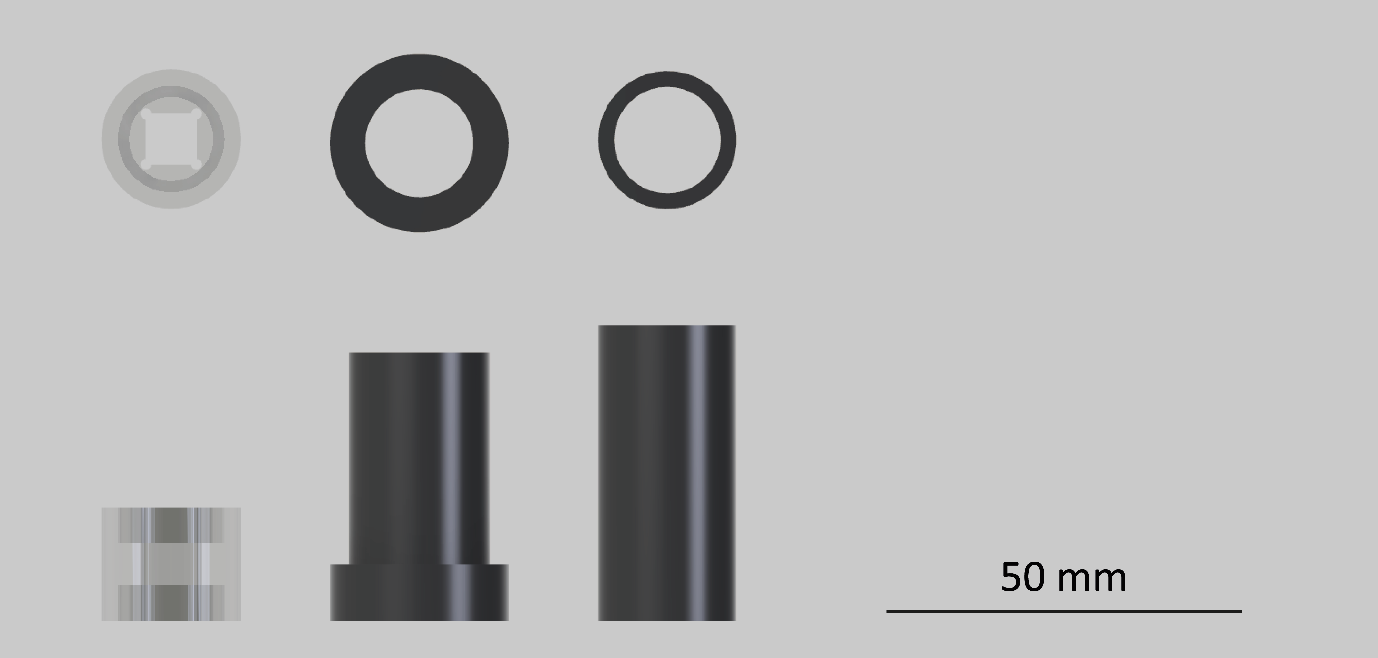


Figure S3. Higher resolution image of components B, C and D in Figure S2.


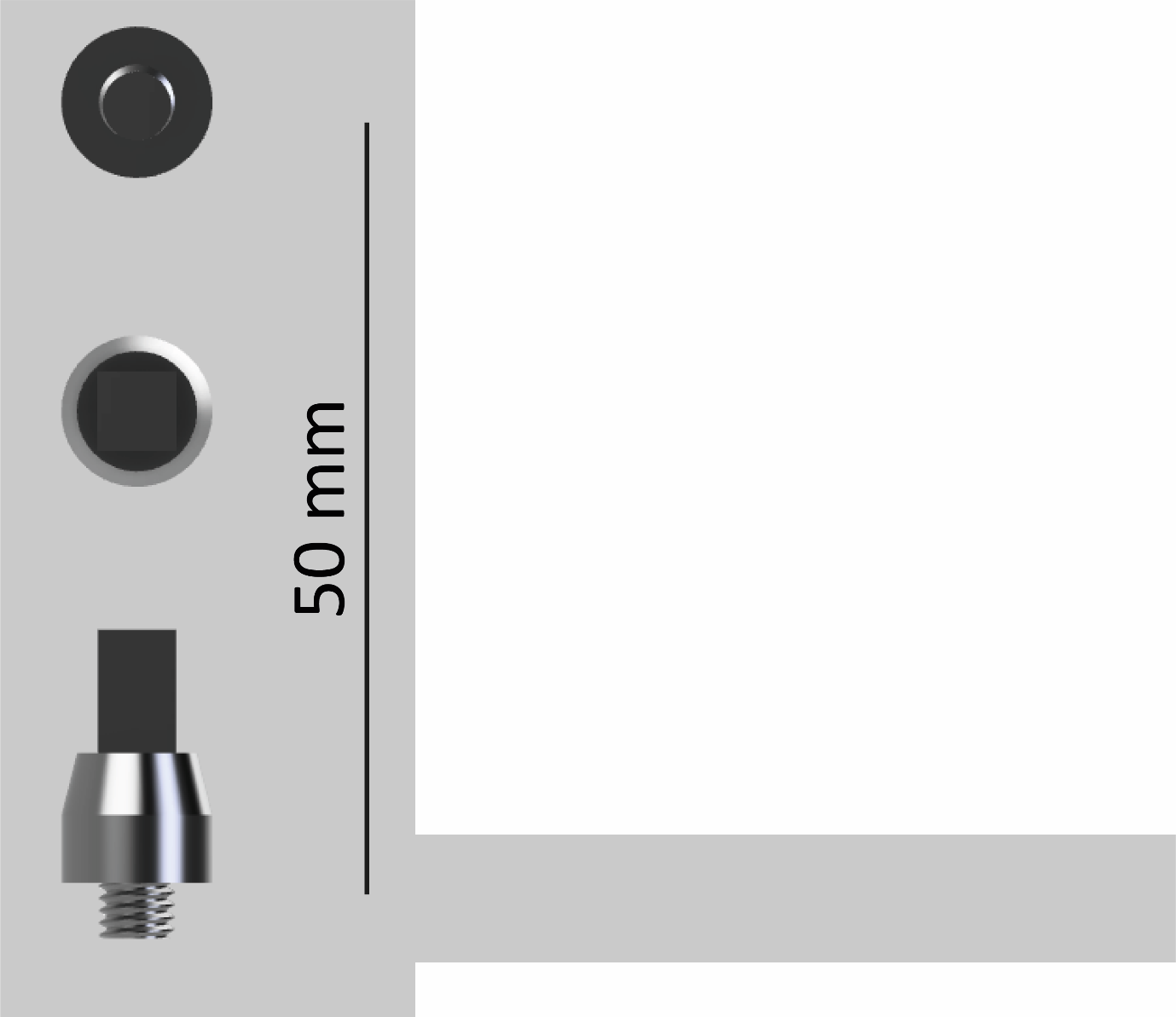


Figure S4. Samples are mounted to the sample transport rod via an M5 tapped component which is inserted within the hollow interior of the transport rod to ensure alignment, this component is shown here with side view (left), from the squared off end (middle) and from the tapped end (right).

While the LF probe fits within the standard Bruker 3-axis gradient system, this latter must be aligned with the HF probe while a guide for the shuttling NMR tube is also provided. This mounting/guiding part of the equipment has been realised by manufacturing the parts shown in Figures S5 to S7.


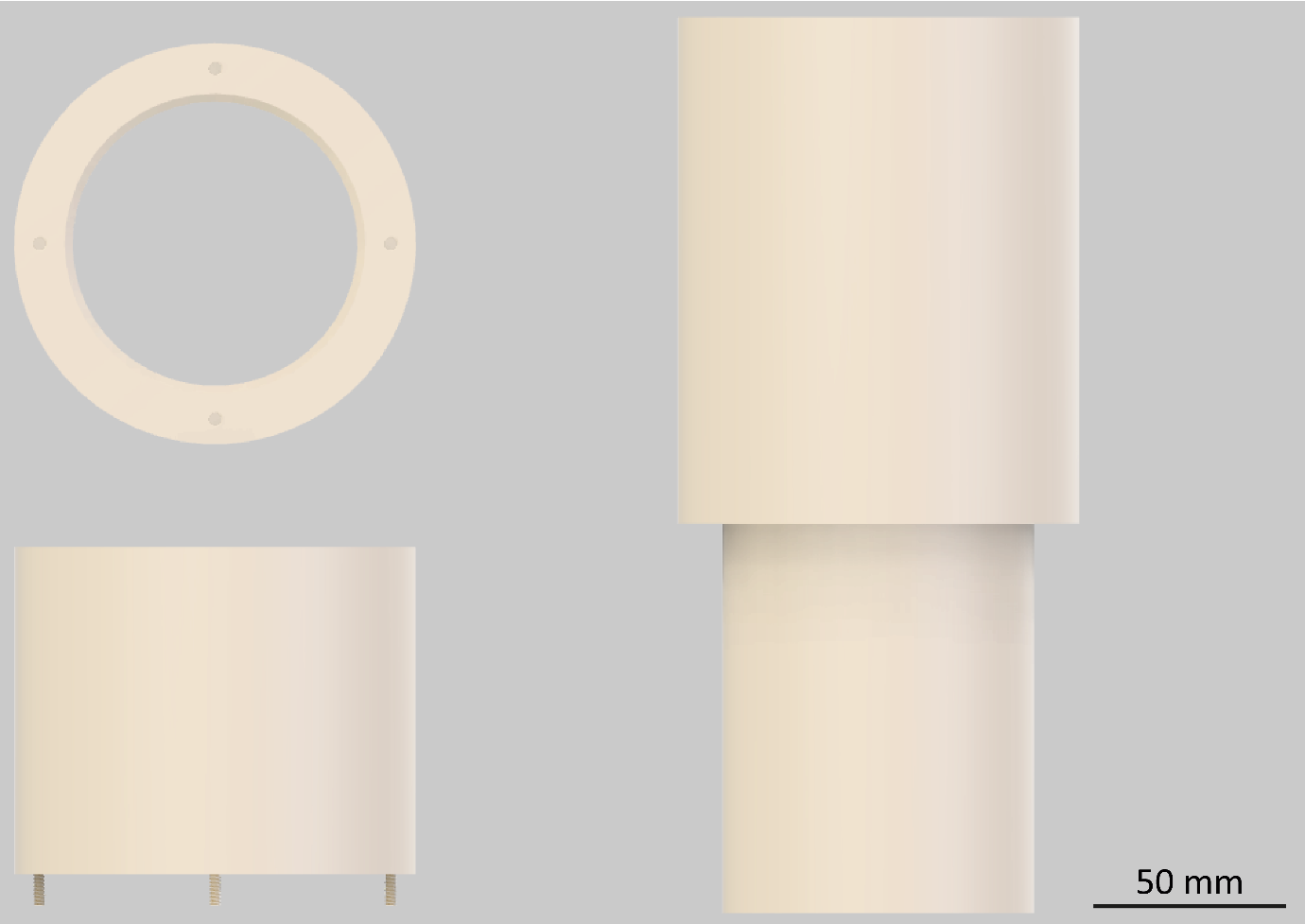
Figure S5. Plastic mountings used to hold the gradient system in place around the LF sweet spot. The mounting is assembled in two parts (A and B) to facilitate the gradient and probe assembling (due to low ceiling height). 4 holes at the top of A engages with the pins at the bottom of B for alignment. 4 holes at the top of B allow the securing of the gradients in the upside-down position (see Figure 2 in main paper).

A

B

C


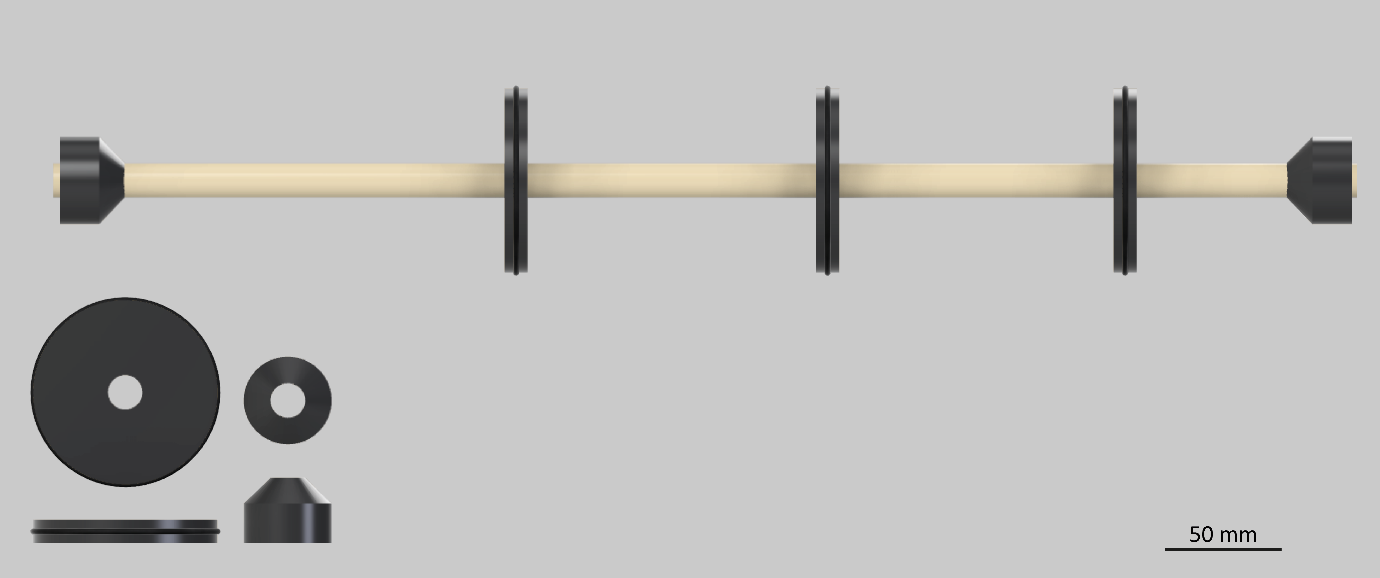
Figure S6. Guidance for the transport rod + sample between the HF and LF is provided by the inter-probe tube guide shown here. Nylon centring disks with o-rings ensure that this sits coaxially within the magnet bore. The two nylon end caps sit tight within the housing of each probe.


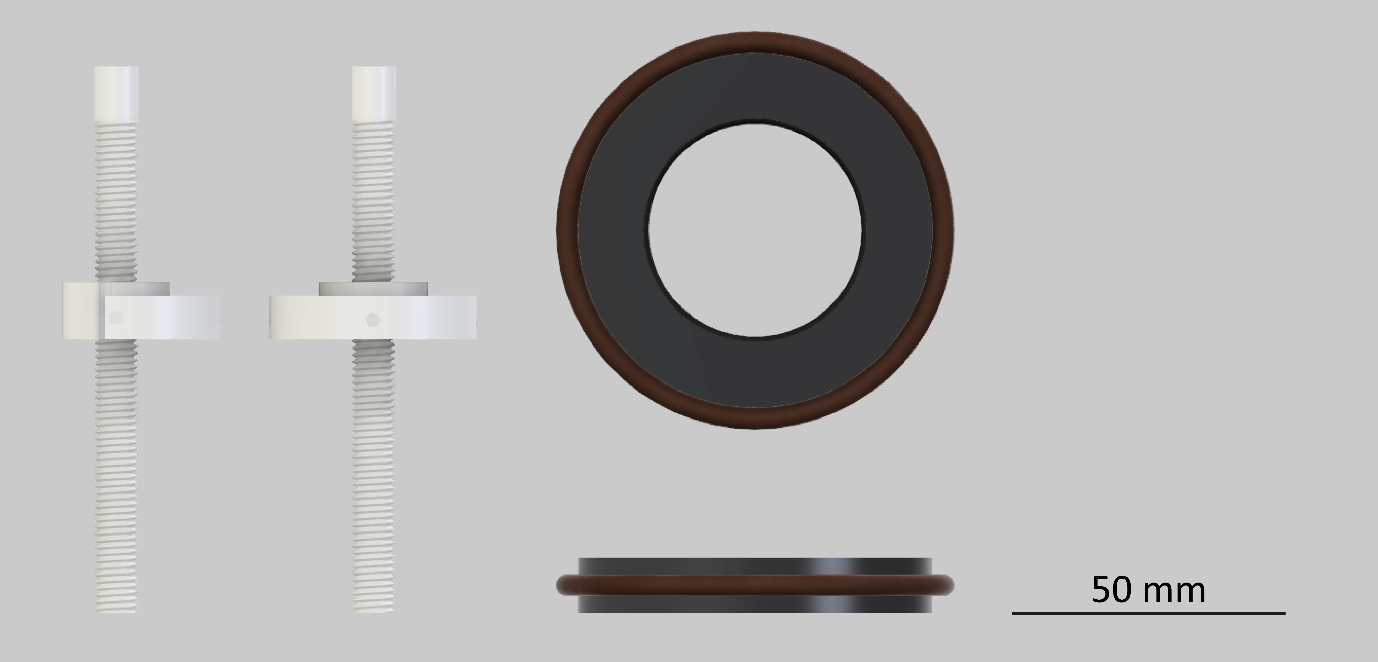


Figure S7. High field probe depth gauge (left) shown from front and side, centring guide for high field probe within magnet bore (right).

## Low field probe power simulations

### Probe Requirements

1. Transmission capability only, no reception

2. Centre frequency ≈ 500 kHz

3. Field inhomogeneity ≈ 30 kHz/cm

4. Inner diameter = 10 mm

5. Homogeneous length = ca. 25 mm

### Reference Power Calculations

#### 3D Field Simulations

Using CST Studio, we first model the coil as a saddle coil with a single winding. Once the inductance is known for a single winding then extrapolation to more windings can be done. The coil is modelled as a wire coil with a wire radius of 0.5mm and a thickness of 0.8mm:


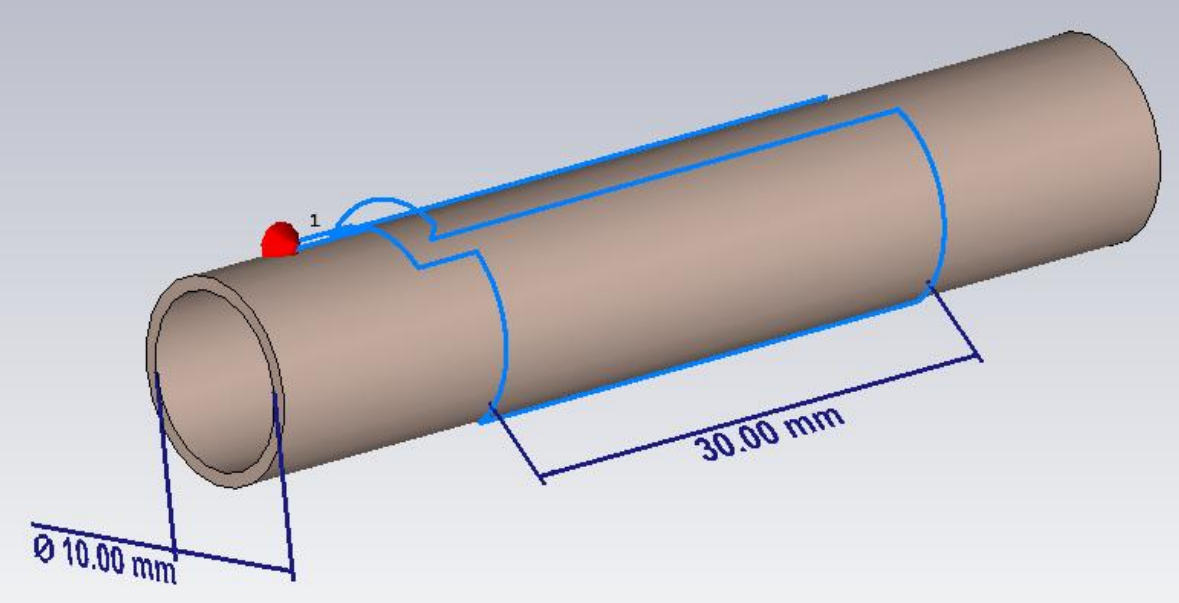


Figure S8. CST Studio model of saddle coil geometry.

By artificially introducing enough losses (resistor Rs), we get perfect matching at the target frequency


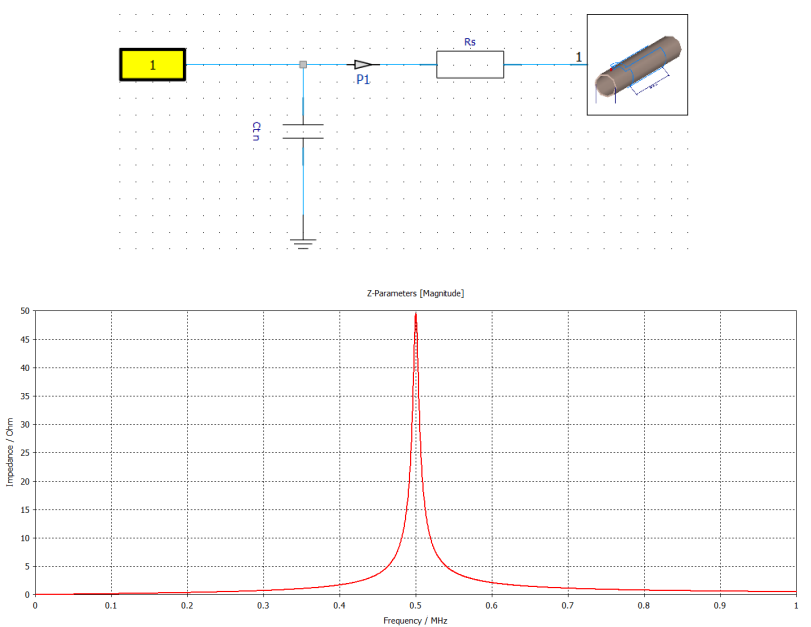


Figure S9. Probe wiring and impedance used for simulations.

The inductance of the single winding can be determined from the impedance at the probe P1 (see Figure S9) and is equal to:

L_coil = 245 nH

Normalized to the accepted power we find a B1 field value equal to:

B1_W_ (centre) = 1.56 mT/√W

and normalized to the current flowing in the coil we find:

B1_A_ (center) = 0.12 mT/A


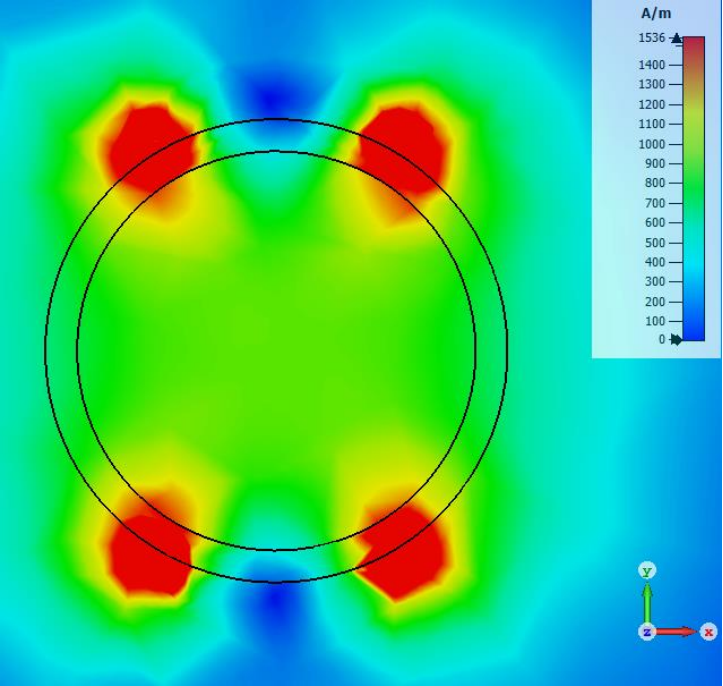


Figure S10. H-field distribution in the centre of coil for 0.5 Watts of accepted power.


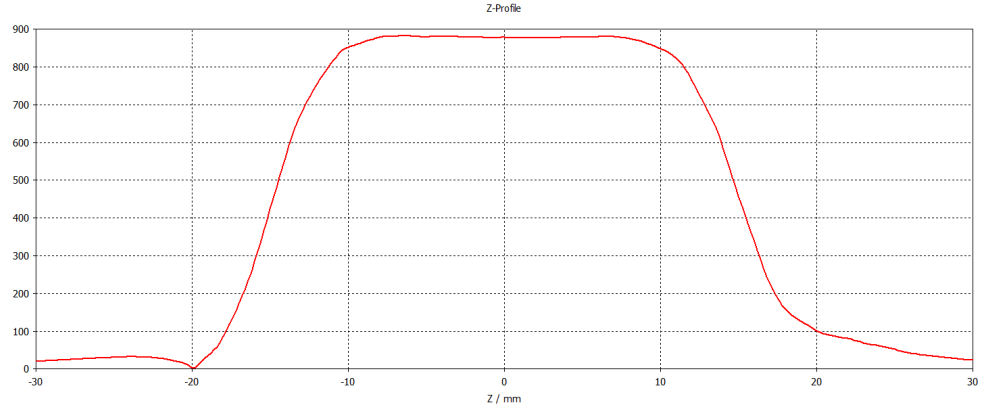


Figure S11. Calculated H-field profile along the z-axis (in A/m).

#### Extrapolation to more windings

Since it’s challenging to model a saddle coil with more windings, we approximated N windings of the same saddle coil by multiplying the inductance of the coil with N^2^. Simplify the system we have then changed the tuning capacitor and the resistor such that we again achieve matching at 500 kHz.
Counterintuitively, this results in a coil that has lower Q the more windings we use, since the resistance to achieve matching scales with N^4^. We then used a pulse length of 10 μs for the excitation signal.


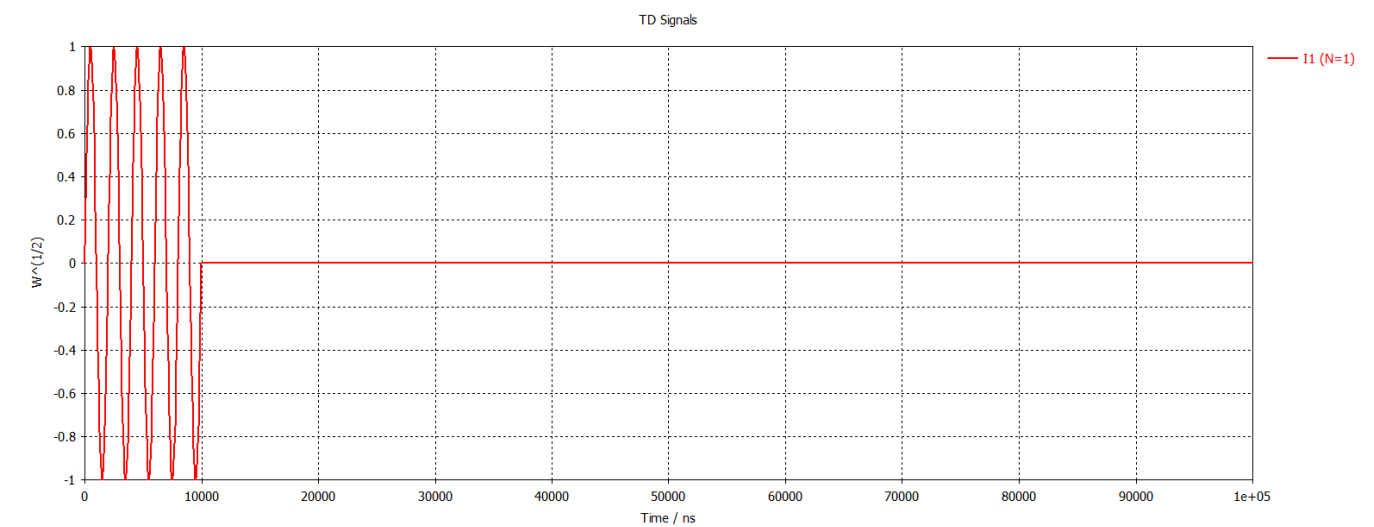


Figure S12. Excitation pulse with length of 10 μs.


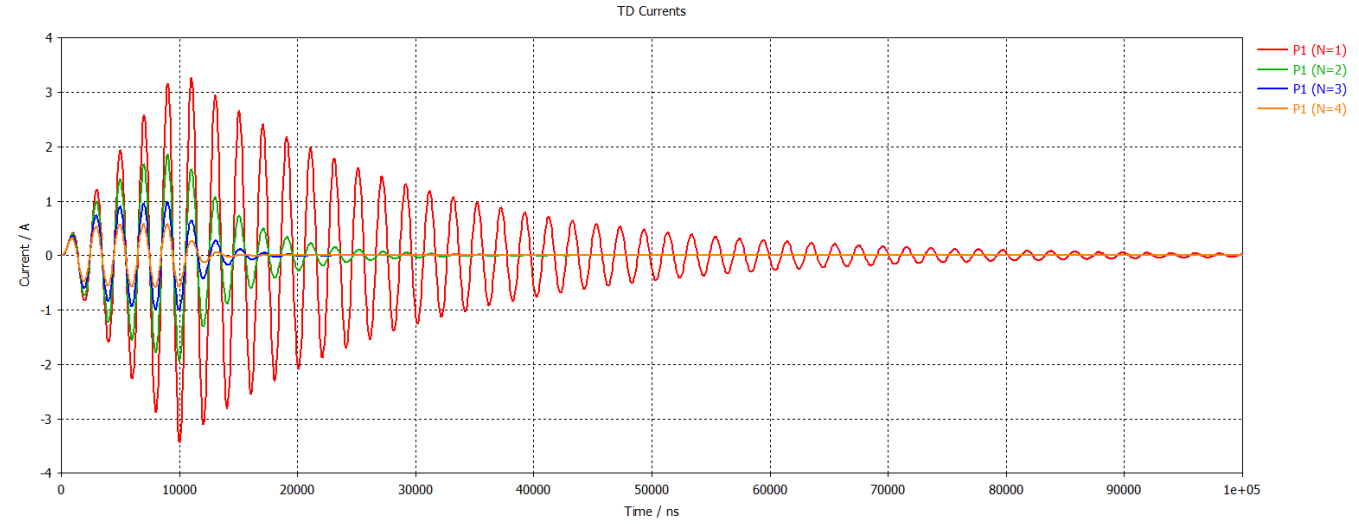


Figure S13. Resulting current in probe coil when the number of windings N is varied.

Scaling the current with the number of windings and using the result for B1_A_ we derived a good estimate for the expected pulse in μT. Since the excitation was always performed with the same amount of power (0.5 Watts), we can furthermore normalize the pulse to 1 Watt of accepted power.


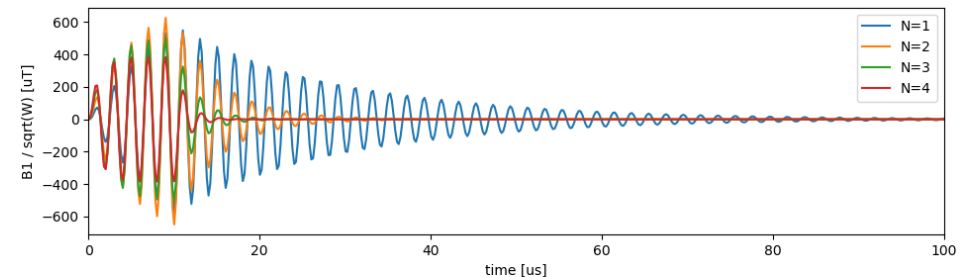


Figure S14. Pulse with variable numbers of coil windings normalised to 1 W accepted power.

In order to get a better estimate of the achievable excitation profile, we also performed a Bloch simulation for the given pulses. Along the probe axis (z-axis) we assume a constant linear gradient of 30 kHz/cm around the centre position, where 500 kHz was assumed, that represents the field inhomogeneity. Furthermore, for simplicity, the field profile along the axis of the coil was not taken into account, i.e. we assume a constant B1 along the whole x-axis of the plot.

Maximum flip angles @ 1 Watt are as follows:

N = 1: 30°

N = 2: 40°

N = 3: 59°

N = 4: 111°


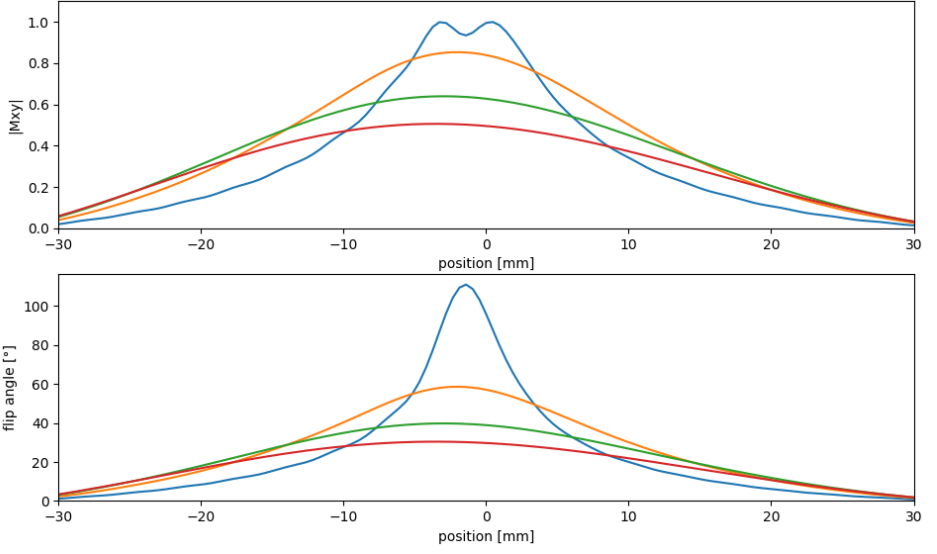


Figure S15. Excited transversal magnetization (top) and corresponding flip angle (bottom).

Note the asymmetry in the excitation profile. A comparison with ideal pulses appears to indicate that this effect can be attributed to the linear operation of the coil and seems to be an effect of the non-negligible B1-minus contribution at such low frequencies (Bloch-Siegert) combined with local field inhomogeneities (see also Figure S16).

The power requirements for 90° pulses are:

N = 1: P ≈ 0.7 W

N = 2: P ≈ 2.4 W

N = 3: P ≈ 5.1 W

N = 4: P ≈ 8.8 W

We have therefore chosen to work with N=4. The pulse shape at the actual wattage to obtain a 90 degrees pulse is shown in Figure S16 (top). The effective flip angle at different field homogeneity corrections (see Table S1 and discussion below) is shown in Figure S16 (bottom). As discussed in the sections below and in Figure S16, shimming the static magnetic field with the use of the Z-shim coil and a current of 3.2 A result in a quite good value of the pulse flip angle along the whole sample.

Figure S16. Final calculated excitation profiles with a given B1 field (top), effective flip angle under various field homogeneity corrections (bottom).

## Low field Temperature

### Probe Temperature Limit Determination

All temperature measurements made use of a Ref-Therm 30 precision plus digital thermometer (serial number D17340735) equipped with a 150mm RTD probe which was mounted to be touching the interior surface of the coil. All measurements took place on a bench as close as possible to the site of the low field probe hardware. For all measurements the low field probe was insulated, and the internal bore blocked with a custom nylon insert to restrict airflow through the bore to obtain a sort of worst-case scenario.

Figure S17. The temperature change recorded at the inner surface of the probe around the probe coil plotted versus time and for different values of the current supplied to the Z-shim coil.

The measurements in Figure S17 show that a current of 3.2 A (the value that best optimise the field homogeneity in LF) results in a heating of ~20 °C which settle down in about 90 min. This corresponds to an absolute temperature of ~43 °C considering the ambient temperature of the room hosting the equipment is regulated to 23 °C. The probe maximum operation temperature is limited to 60 °C due to the nature of the materials used for its construction. In principle the Z-coil can therefore support a current of up to 4.25 A as can be extrapolated by the plot in Figure S18.

Figure S18. Plot of the temperature increase after 80 mins from the application of current into the shim coil against the current applied, orange line guides the eye to the predicted hardware temperature limit of 60 °C.

### Effect of Shim coil Heating on Sample Temperature

Finally, we sought to determine the effect that the increased temperature of the inner LF probe surface has on the actual NMR sample (with all parts mounted and moving as in an actual experiment). For this we used a sample of ethylene glycol (≥ 99%, **S4**) prepared inside a modified 10 mm tube identical to that described for **S3**. Ethylene glycol is typically used to calibrate temperature in NMR. In the interval ~300K-420K the sample temperature can be obtained through the equation^1^:

$$T=\frac{(4.637- \Delta\delta)}{0.009967}$$

Equation S1. Calculation used for determination of temperature from sample **S4**.

where $\Delta\delta$ is the distance, in ppm, between the CH2 and OH peaks of ethylene glycol. Figure S10 shows the temperature of **S4** as a function of the current in the Z-shim coil. To obtain these data the sample is kept in HF for 2h for each value of the Z-shim coil current. This is to make sure that the LF probe reaches a stable temperature at the value generated by the current chosen. The sample is then shuttled to LF and left there for 10 minutes. After that, the sample is shuttled back to HF and a 90-acq pulse sequence is used to acquire a 1H-NMR spectrum from which $\Delta\delta$ is obtained.

Figure S19. The temperature measured in HF on **S4** after 10 mins spent in LF at different values of the current in the Z-shim coil. Note that the HF probe is at ~25 °C (probably due to the heating produced in the bore by the room temperature shims) while the ambient temperature is about 23 °C hence the base temperature of the HF probe and the LF with Z-shim off are actually different.

From Figure S19 note that, at a current of 3.2 A (the value corresponding to the best field homogeneity around the LF sweet-spot - see Figure 3 in the main paper) the actual sample temperature raises to 34.2 °C, in the conditions used for these experiments.

However, in the experiments presented in this paper the longest time the sample is kept in LF is 2 minutes. To simulate the most relevant worst-case scenario we repeated the temperature measurement above, still using **S4**, but this time with the sample spending 3 minutes in HF and 2 minutes in LF but repeating the measurements 8 times to simulate a multiple-scan data acquisition. The result of this experiments is reported in Figure S20.

Figure S20. Plot of the temperature change measured on **S4** during the progression of measurement of diffusion coefficient along the z direction with a 120 s diffusion time ∆ and a 3.2 A shim correction. Measurements were taken immediately following the return to HF after 2 mins at LF, the sample then remained at HF for 3 mins before returning to LF for the next scan.

Figure S20 shows that over a number of repeats the sample reaches a fairly constant and repeatable temperature of (30.45 ± 0.47) °C. This is to say that all diffusion measurements done with our apparatus and reported in this paper refer to a temperature value of ~30 °C.

To mitigate this heating problem, we have programmed the Z-shim coil to be *on* during the pulse sequence and *off* while the sample is polarising in HF or during the diffusion time in LF in the case of a diffusion experiment.

## Shim Measurements

Initial manual measurements of field correction were taken with the LF probe in situ making use of a Lakeshore 460 3-channel gaussmeter equipped with a MZ-2518-UH hall probe. These measurements have a spatial-positioning error of about ±1 mm and indicate that a 3 A current in the Z-shim coil provides a relatively good main field homogeneity in the LF coil region. This figure is only indicative and was used as a ballpark in finding the experimentally determined value of 3.2 A found using the procedure explained in the main paper (Figure 3b, main paper).

Table S1: Magnetic field measured across the LF probe coil around the low field sweet spot field of 46.4 mT and as a function of the current applied to the Z-shim coil. A positive value of the distance refers to the region above the LF sweet spot.

|  | Current in shim coil (A) | | | | | | | | | | | | |
| --- | --- | --- | --- | --- | --- | --- | --- | --- | --- | --- | --- | --- | --- |
|  | 0 | 0.25 | 0.5 | 0.75 | 1.0 | 1.25 | 1.5 | 1.75 | 2.0 | 2.25 | 2.5 | 2.75 | 3 |
| Distance (mm) | Magnetic Field (mT) | | | | | | | | | | | | |
| 10 | 44.1 | 44.3 | 44.5 | 44.7 | 44.9 | 45.1 | 45.3 | 45.5 | 45.7 | 45.9 | 46.1 | 46.3 | 46.5 |
| 5 | 45.3 | 45.4 | 45.5 | 45.6 | 45.7 | 45.8 | 45.9 | 46.0 | 46.1 | 46.2 | 46.3 | 46.4 | 46.5 |
| 0 | 46.4 | 46.5 | 46.5 | 46.5 | 46.5 | 46.5 | 46.5 | 46.5 | 46.5 | 46.5 | 46.5 | 46.5 | 46.5 |
| -5 | 47.7 | 47.6 | 47.5 | 47.4 | 47.3 | 47.2 | 47.1 | 47.0 | 46.9 | 46.8 | 46.7 | 46.6 | 46.5 |
| -10 | 48.9 | 48.7 | 48.5 | 48.3 | 48.1 | 48.0 | 47.8 | 47.6 | 47.4 | 47.2 | 47.1 | 46.4 | 46.2 |

# Chemical Synthesis

## General information

All reactions were carried out under a positive pressure of argon using inert atmosphere methods and in oven-dried glassware unless otherwise stated.

## Solvents and reagents

All chemicals used were purchased from Merck, Alfa Aesar, Thermofischer Scientific or Fluorochem and used without further purification. Anhydrous and deuterated solvents were purchased from Merck or Fluorochem and were used as supplied.

## Chromatography and equipment

Analytical t.l.c was performed using Merck Kieselgel 60 F254 (230-400 mesh) fluorescent treated silica plates. Visualisation was carried out using UV radiation (254 nm) and aqueous potassium permanganate solution unless otherwise stated. Flash column chromatography was carried out using Merck Kiesegel (230-400 mesh).

## Analytical techniques

^1^H and ^13^C spectra were measured using a Bruker AVII400 or AVIIIHD400 FT NMR spectrometer. ^1^H NMR spectra were measured at 400 MHz. ^13^C spectra were measured at 100 MHz. Topspin™ software was used for spectral interpretation. All chemical shifts are quoted on the δ scale using deuterated chloroform as the internal standard unless otherwise stated.

^1^H NMR spectra are reported as follows: ^1^H NMR (spectrometer frequency, solvent) ppm
(number of protons, multiplicity, J coupling constant where applicable, assignment). ^13^C NMR spectra are reported as follows: ^13^C{^1^H} NMR (spectrometer frequency, solvent) ppm (multiplicity, J coupling constant where applicable, assignment). Coupling constants (J) are given in Hertz to the nearest 0.1 Hz and chemical shifts (δ) are given in parts per million. Multiplicity of signals are denoted as follows: s = singlet, d = doublet, t = triplet, q= quartet, dd= doublets of doublets, dt = doublet of triplet etc. Assignments of protons refer to the corresponding figures and may not agree with the IUPAC names. Peaks that could not be assigned are denoted ‘Ar-H’ or ‘CH-Ar’. All chemical shifts (δ) are quoted using a residual solvent peak as the internal standard. All NMR data was recorded at 298 K unless otherwise stated.

ChemDraw (CambridgeSoft™) professional software was used to generate electronic structures of compounds and systematic names.

IR spectra were obtained using the Thermo Scientific Nicolet™ iS™ 5 with a diamond ATR module and 16 scans per spectrum in the region 500 – 4000 cm^-1^ with a resolution of 4 wavenumbers per cm^-1^. Absorption maxima (ν_max_) reported in wavenumbers (cm^-1^).

High- and low-resolution mass measurements were undertaken at the University of Southampton using electron impact (EI) techniques. Gas Chromatography-mass spectrometry (GC-MS) electron/chemical ionisation (EI/CI) used a Thermo (Hemel Hempstead, UK) Trace GC-MS single quadrupolar mass spectrometer.

Melting points were recorded using a Stuart (Bibby Scientific Limited) SMP20 machine and are uncorrected.

## Synthetic Scheme

Scheme S1. Synthetic scheme for 1,2-Diphenylethyne-1,2-^13^*C*_2_.

### Trimethyl(phenylethynyl-1,2-^13^*C*_2_)silane **II**

Iodobenzene **I** (0.16 mL, 1.47 mmol), tetrakis(triphenylphosphine)palladium(0), (85.0 mg, 0.07 mmol) and copper(i) iodide (28.0 mg, 0.14 mmol) were added to a microwave vial and sealed. The vial was evacuated and back filled with argon before triethylamine (3.00 mL) was added. The solution was degassed with a stream of argon before (trimethylsilyl)acetylene-^13^C_2_ (0.22 mL, 1.54 mmol) was added dropwise. The reaction was heated to 80 ˚C for 1.5 h. The solution was purified by flash column chromatography (petrol) to give the *title compound* **II** (255 mg, 1.45 mmol, 99%) as a colourless liquid; R*_f_*  0.70 (petrol). δ_H_ (400 MHz, CDCl_3_) 7.52-7.44 (2H, m, Ar-H), 7.35-7.27 (3H, m, Ar-H), 0.27 (9H, d, *J* 2.7, (CH_3_)_3_); δ_C_ (100 MHz, CDCl_3_) 132.1 (t, *J* 2.2, C2), 128.6 (s, C4), 128.3 (d, *J* 5.2, C3), 123.2 (dd, *J* 84.1, 9.8, C1), 105.3 (d, *J* 135.6, ^13^C-enriched, C≡C), 94.1 (d, *J* 135.6, ^13^C-enriched, C≡C), 0.13 (dd, *J* 5.2, 1.4, (CH_3_)_3_); IR (neat) ν_max_ 2958, 2079, 1487, 1443, 1248, 1212, 1026, 856, 836, 753, 688, 641, 529 cm^–1^; HRMS (EI) calculated for C_9_^13^C_2_H_14_Si [M]^+^ 176.0926, found 176.0923.

### 1,2-Diphenylethyne-1,2-^13^*C*_2_ **III**

Trimethyl(phenylethynyl-1,2-^13^*C*_2_)silane **II** (50.0 mg, 0.28 mmol), tris(dibenzylideneacetone)dipalladium(0) (13.0 mg, 0.01 mmol), triphenylphosphine (15.0 mg, 0.05 mmol), copper(i) iodide (6.00 mg, 0.03 mmol) and iodobenzene **I** (0.28 mL, 2.49 mmol) were added to a microwave vial and sealed. Triethylamine (1.50 mL) and tetrahydrofuran (0.5 mL) were added, and the solution degassed with a stream of argon. Tetra-*n*-butylammonium fluoride (1.0 M in tetrahydrofuran, 2.84 mL, 2.84 mmol) was added and the solution heated to 50 ˚C for 4 h. The solution was concentrated *in vacuo* and the residue purified by flash column chromatography (petrol) to give the *title compound* **III** (25.0 mg, 0.14 mmol, 50%) as a white crystalline solid; R*_f_*  0.50 (petrol); m.p. 58-60 ˚C (petrol). δ_H_ (400 MHz, CDCl_3_) 7.58-7.52 (4H, m, Ar-H), 7.39-7.31 (6H, m, Ar-H); δ_C_ (100 MHz, CDCl_3_) 131.8 (t, *J* 2.2, C2), 128.5 (t, *J* 2.8, C3), 128.4 (s, C4), 123.4 (t, *J* 52.7, C1), 89.5 (s, ^13^C-enriched, C≡C); IR (neat) ν_max_  3064, 1599, 1490, 1442, 1280, 1070, 1025, 917, 752, 687, 528 cm^–1^; HRMS (EI) calculated for C_12_^13^C_2_H_10_ [M]^+^ 180.0840, found 180.0841.

## Spectra


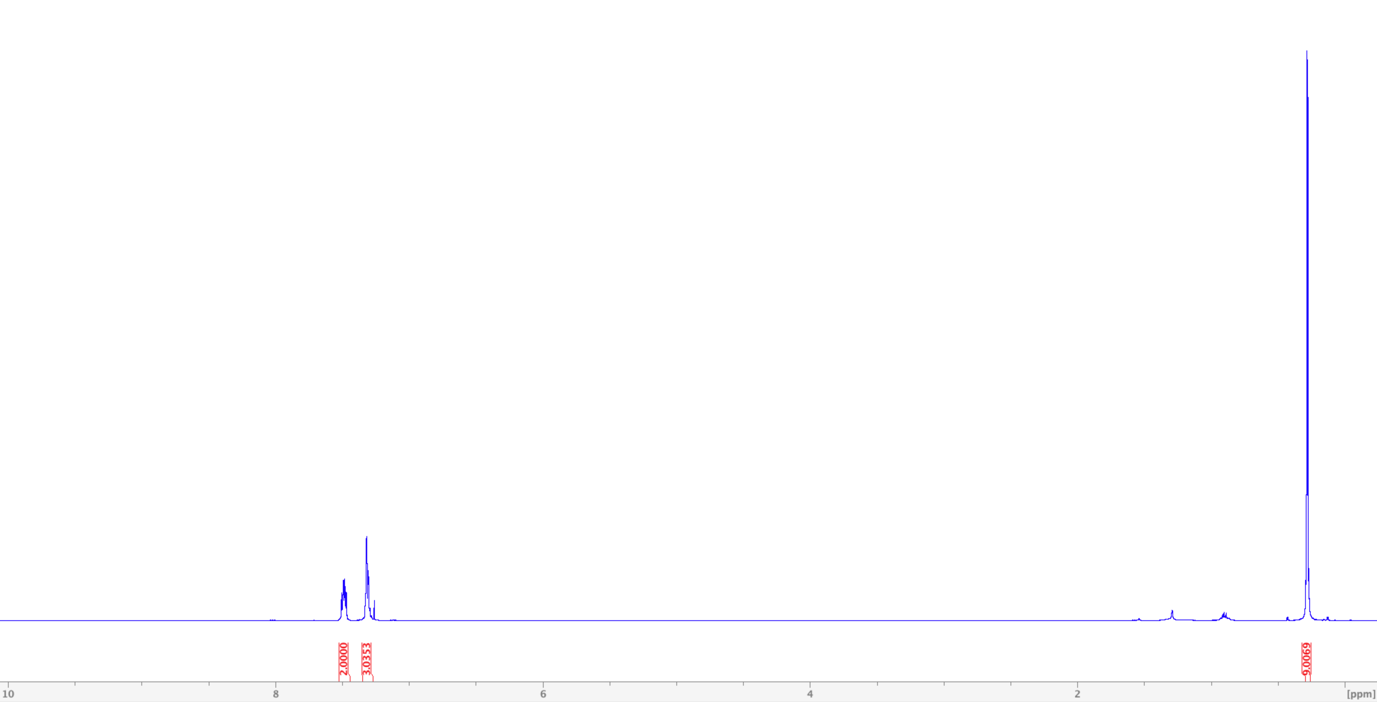


Figure S21. ^1^H NMR spectra of Trimethyl(phenylethynyl-1,2-^13^C_2_)silane.

^
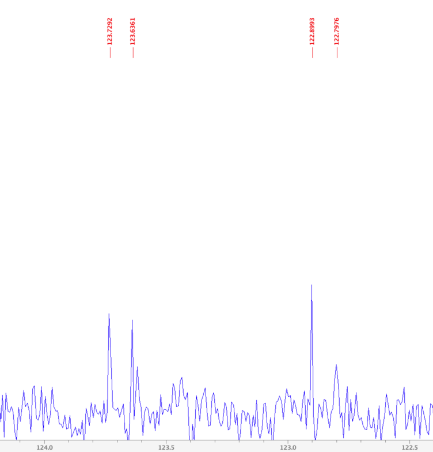
^
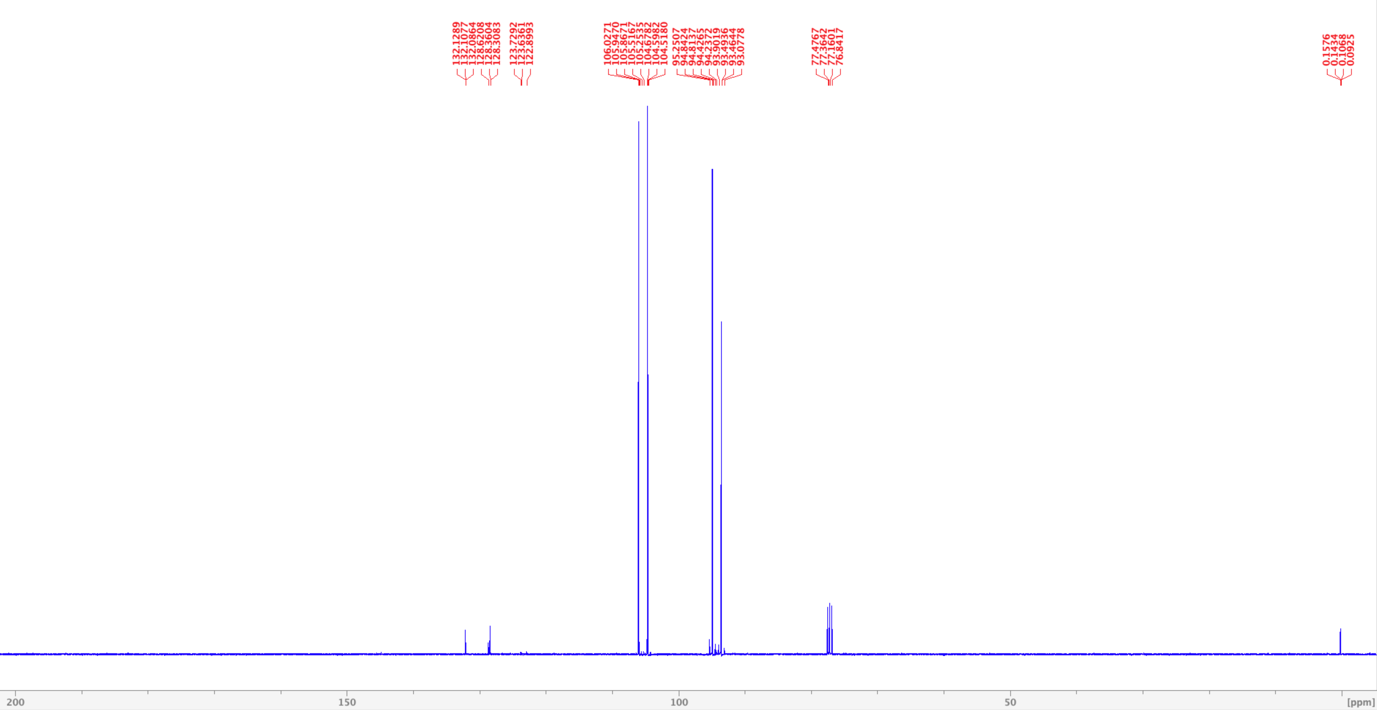


Figure S22. ^13^C NMR spectra of Trimethyl(phenylethynyl-1,2-^13^C_2_)silane with the region between ~122 and ~125 ppm shown as inset.

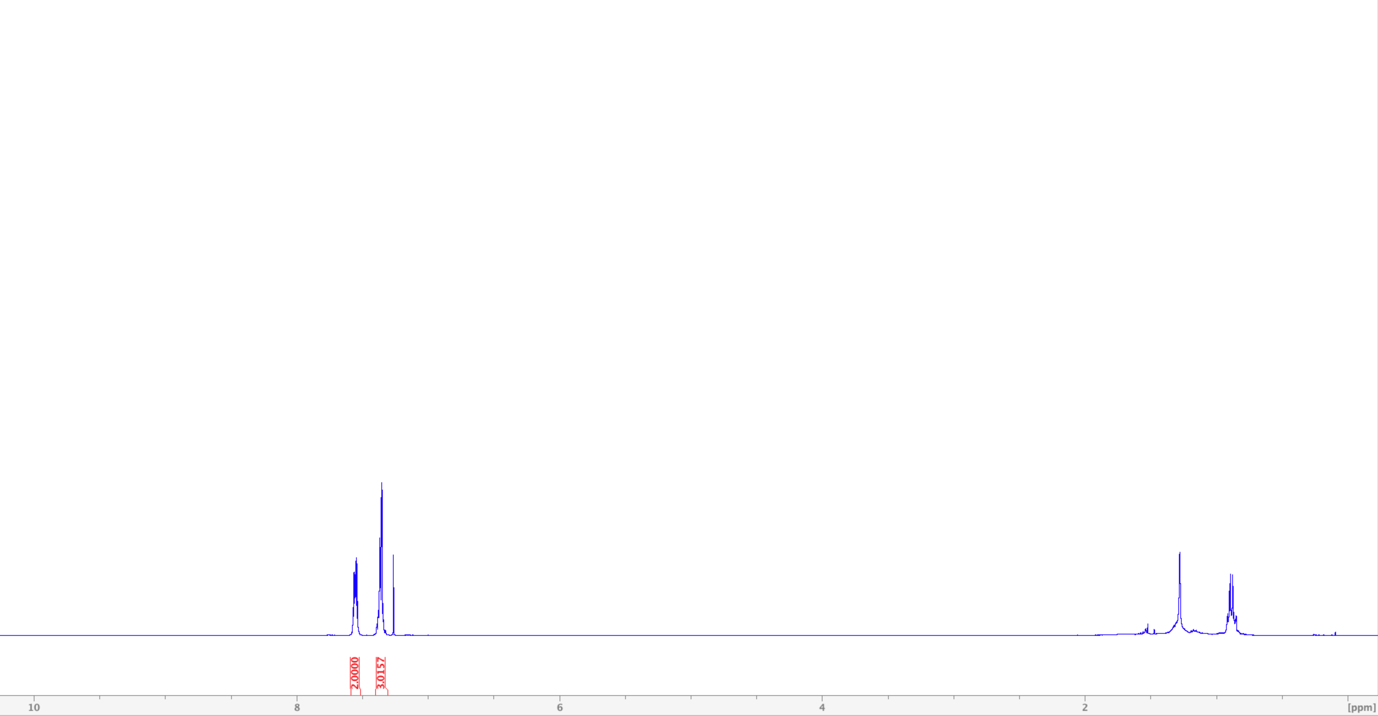


Figure S23. ^1^H NMR spectra of 1,2-Diphenylethyne-1,2-^13^C_2_ **III**.

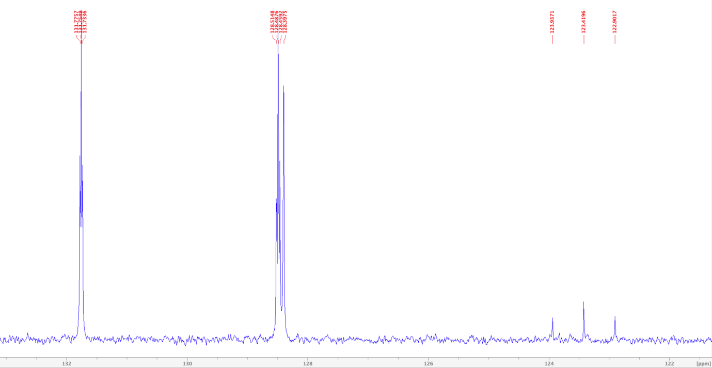
**
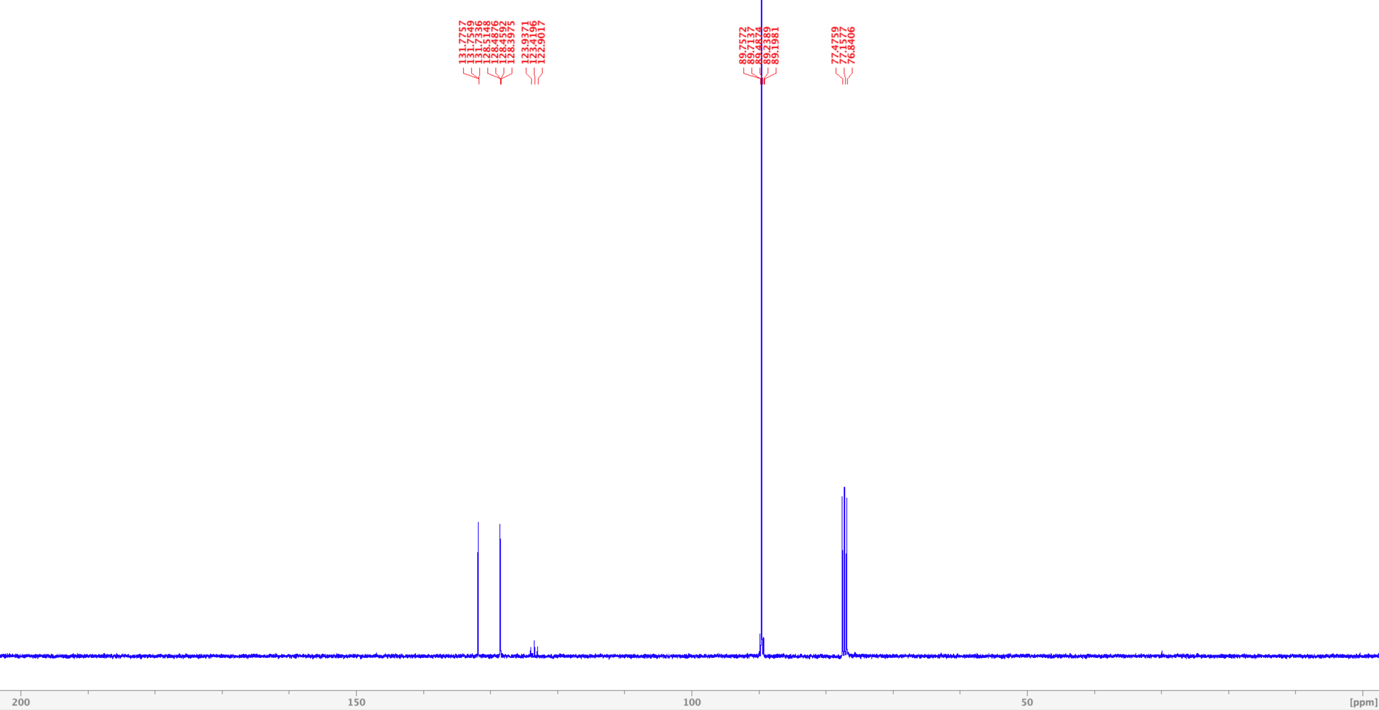
**Figure S24. ^13^C NMR spectra of 1,2-Diphenylethyne-1,2-^13^C_2_ **III** with the region between ~121 and ~132 ppm shown as inset.

**
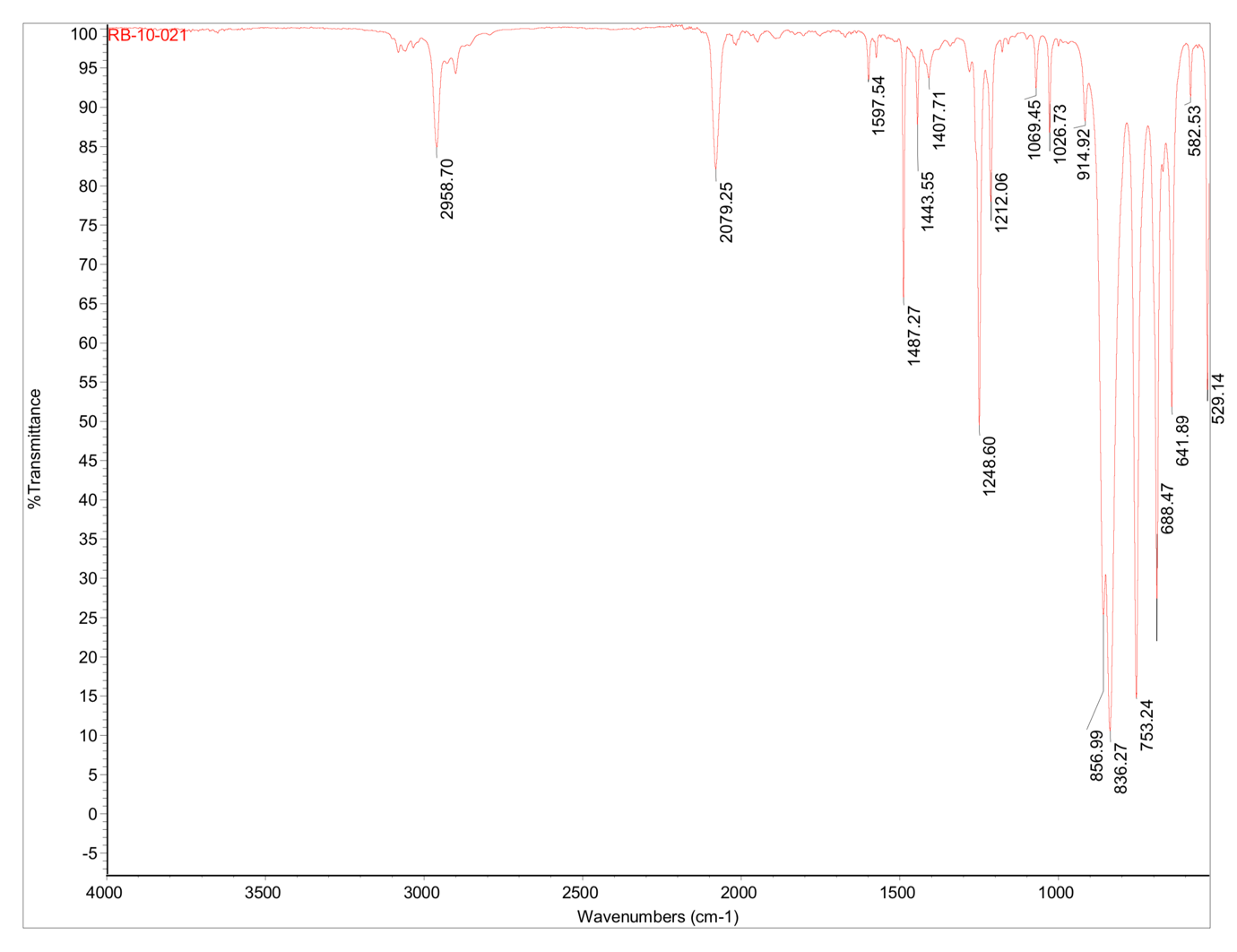
**

Figure S25. IR spectra of Trimethyl(phenylethynyl-1,2-^13^C_2_)silane **II**.

IR spectra of 1,2-Diphenylethyne-1,2-^13^C_2_ **III**

**
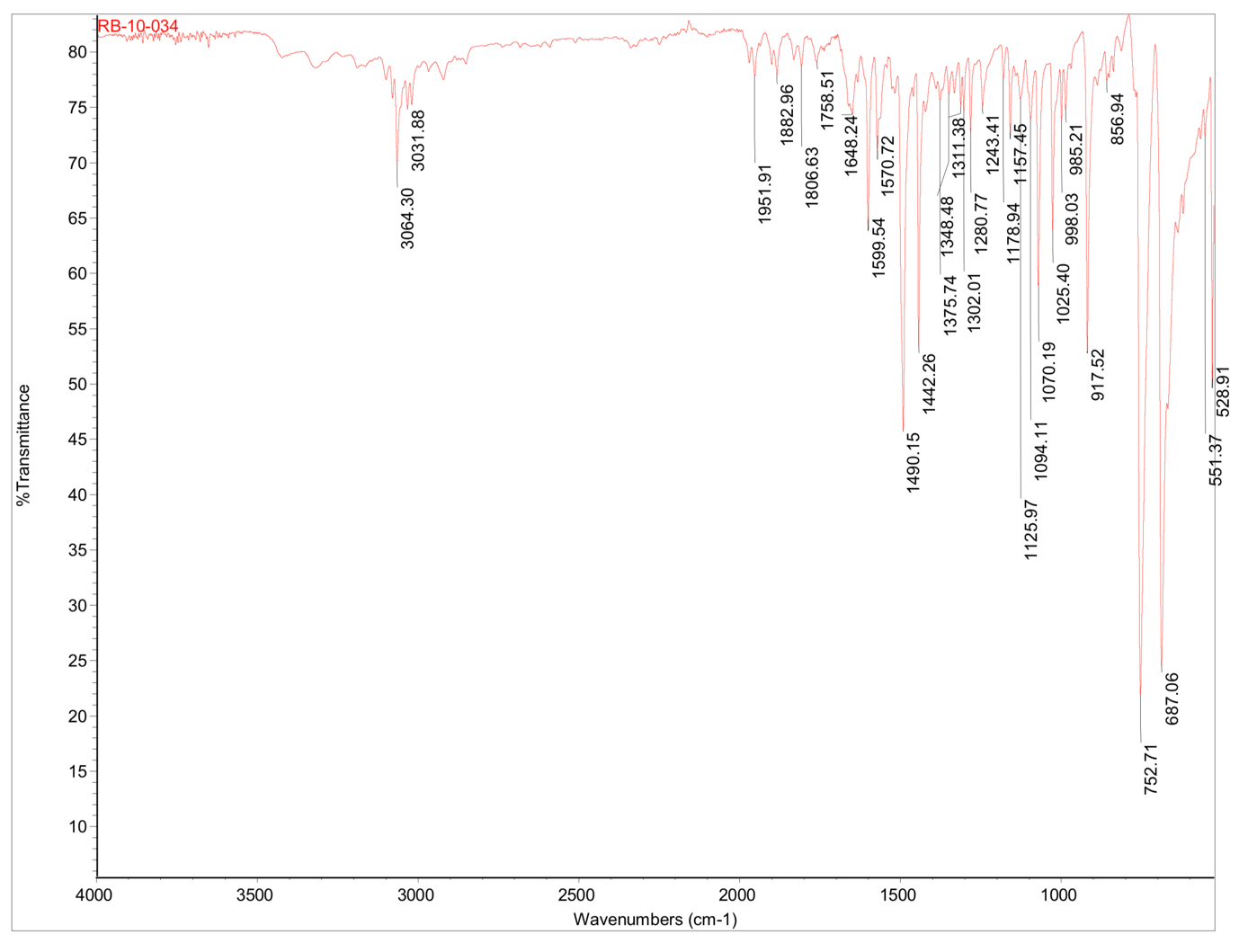
**Figure S26. IR spectra of 1,2-Diphenylethyne-1,2-^13^C_2_ **III**.

HRMS spectra of Trimethyl(phenylethynyl-1,2-^13^C_2_)silane **II**

**
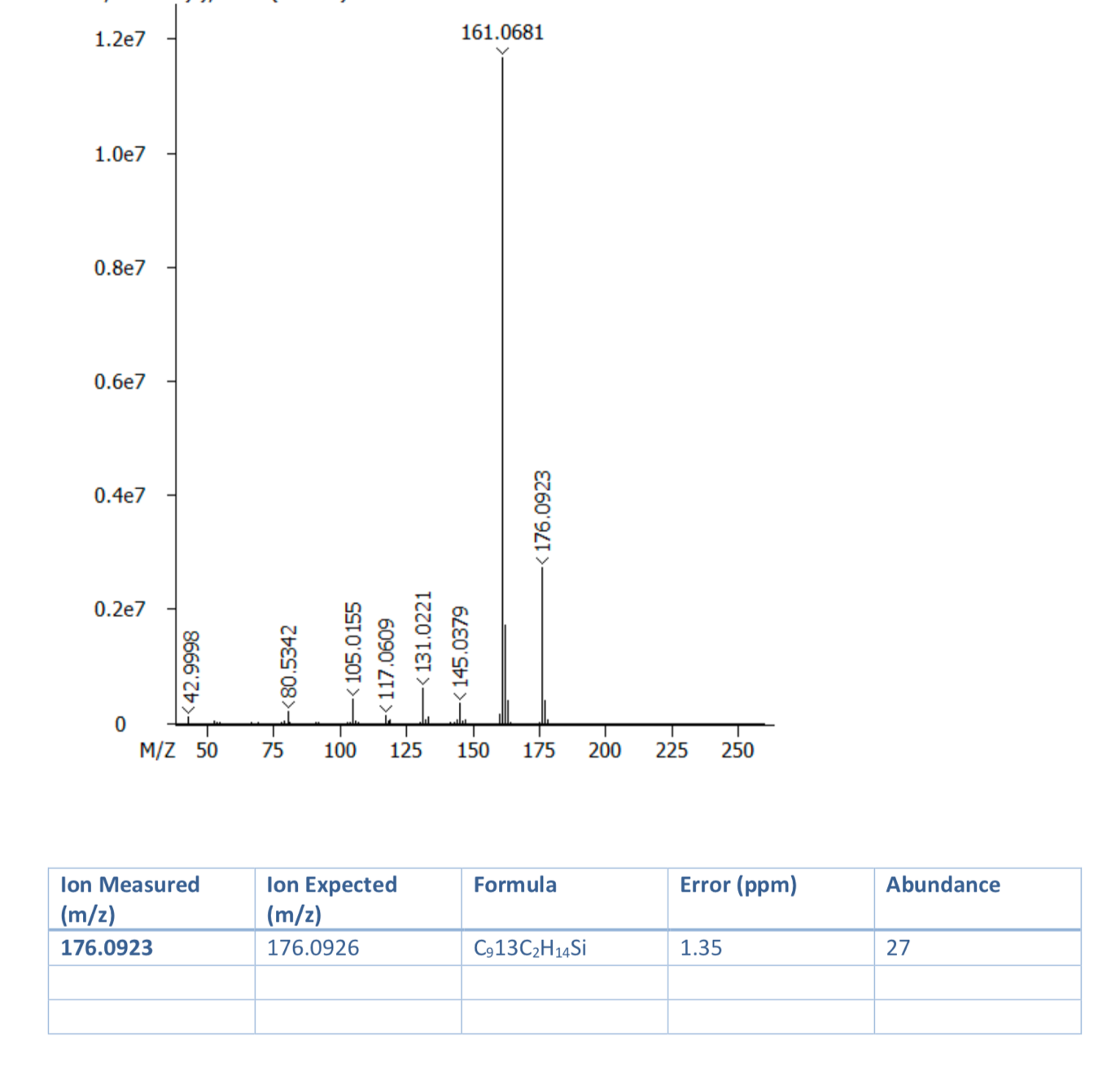
**

Figure S27. HRMS spectra of Trimethyl(phenylethynyl-1,2-^13^C_2_)silane **II.**


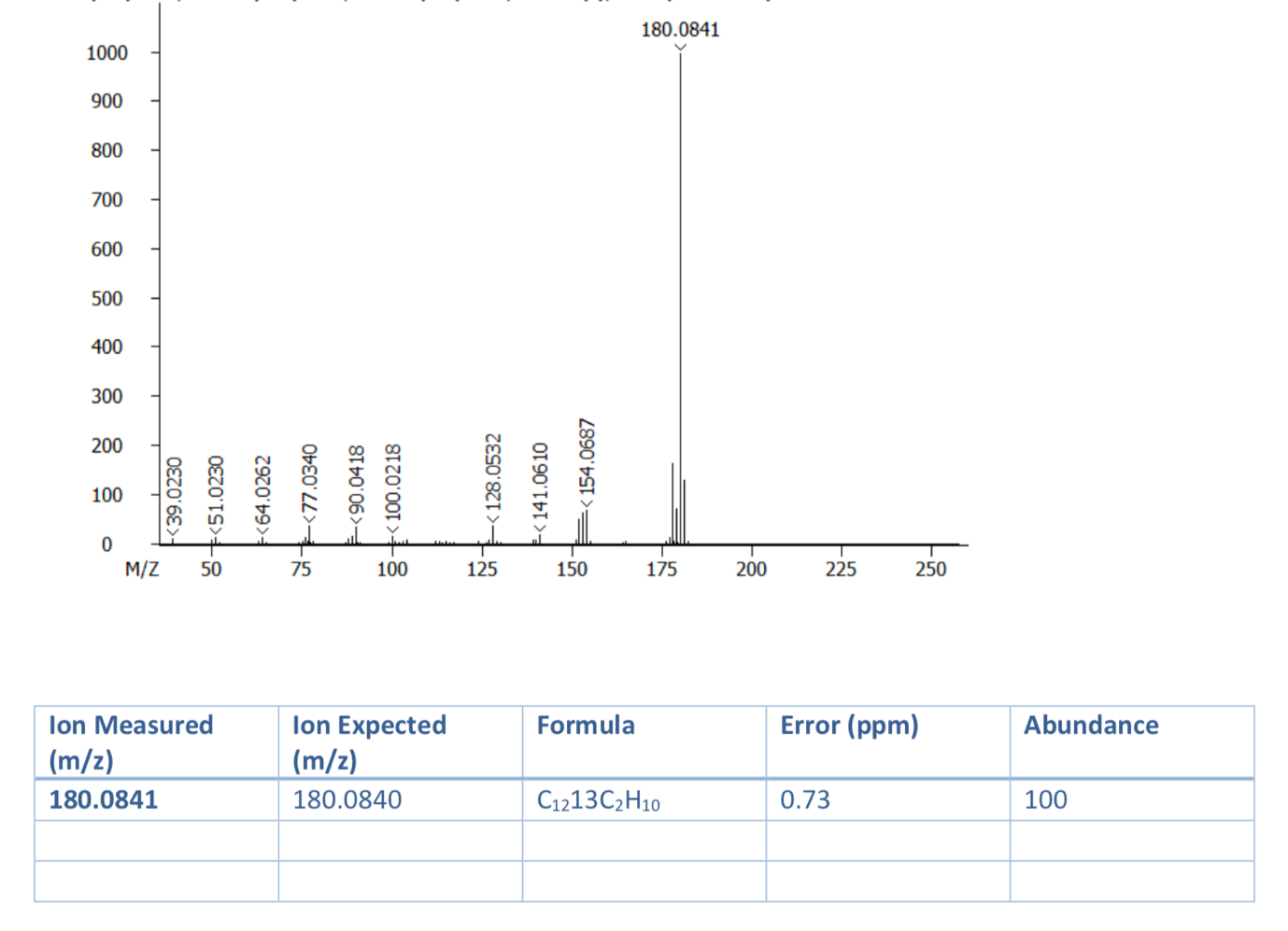


Figure S28. HRMS spectra of 1,2-Diphenylethyne-1,2-^13^C_2_ **III.**

# Single Echo Simulations

Figure S20 shows a simulation of the single echo field-cycled experiment (see Figure 3a of the main paper) done according to the methodology published in Ref. 2. In those simulations a molecule is placed in the center of a 20 mm x 20 mm x 20 mm cubic box and let it take 10^7^ random steps. Each step is chosen according to a Gaussian distribution of zero mean and standard deviation $\sigma=\sqrt{2D_{0}t_{s}}$, with $D_{0}=6.4 x {10}^{-10}$ m^2^ s^-1^ (the measured diffusion coefficient of sample **S1**) and the times step $t_{s}$ duration set to 0.1 ms. In the box there is a linear magnetic field gradient that is built from the experimental measurements reported in Table S1 at the corresponding value of the current in the coil.

Figure S29. Numerical simulation of the single-echo experiment in Figure 3a of main paper. The simulations refer to molecular diffusion in a region of space where there is a linear gradient in the z-direction whose intensity was experimentally measured as reported in Table S1.

The result of these simulations indicates that without a Z-shim coil the field inhomogeneities are quite large and would lead to a rapid decay of the magnetisation. Transverse magnetization can be observed for longer as a current is passed to the Z-shim coil to obtain a better field homogeneity. At 3 A there is no predicted losses of magnetization for a total echo time of the order of 100 ms.

# Sample Spectra Comparison

Figure S30 shows the comparison of the spectra obtained for S2 (a) and S3 (b) in a single scan to appreciate the line broadening due to the presence of the beads in S3. The spectrum in c is the one obtained on S3 the pulse sequence in Figure 9a of the main paper using τ_v_ = 1 s and summing up 16 scans.


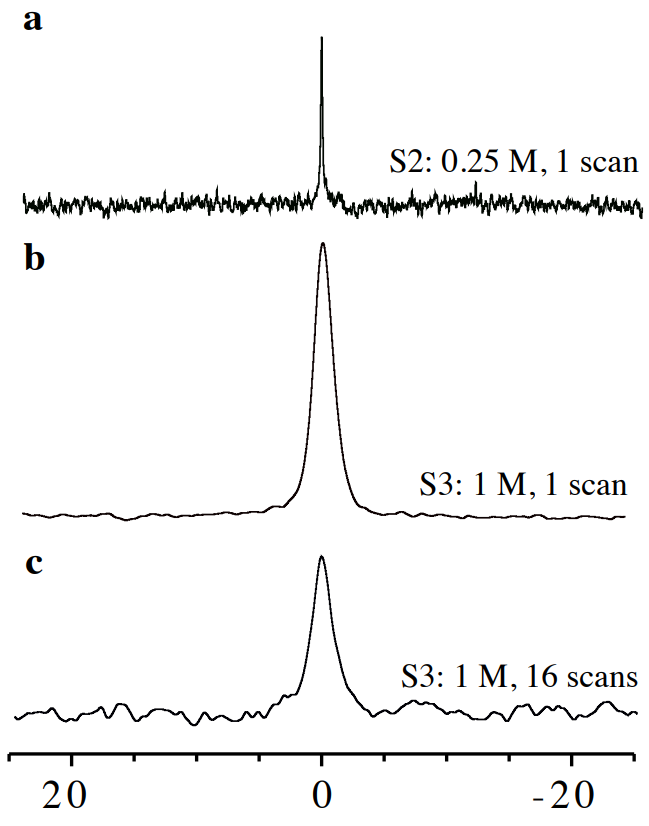


Figure S30. a) ^13^C-NMR spectrum of **S2** at 0.25M concentration. b) ^13^C-NMR spectrum of **S3** at 1M concentration. c) ^13^C-NMR spectrum of **S3** at 1M concentration resulting from the pulse sequence in Figure 9a (main paper) for τ_v_ = 1 s and summing up 16 scans.

# References

1. Bruker Variable Temperature Unit User Manual VTU, http://www2.chem.uic.edu/nmr/downloads/BASHCD10/pdf/z31482.pdf, (accessed May 2023).
2. Cartlidge, T. A. A.; Robertson, T. B. R.; Utz, M.; Pileio, G. Theory and Simulation Framework for the Relaxation of Nuclear Spin Order in Porous Media. The Journal of Physical Chemistry B, **2022**, 126, 6536–6546
